# Induction of Cancer Stem Cell Properties in Colon Cancer Cells by Defined Factors

**DOI:** 10.1371/journal.pone.0101735

**Published:** 2014-07-09

**Authors:** Nobu Oshima, Yasuhiro Yamada, Satoshi Nagayama, Kenji Kawada, Suguru Hasegawa, Hiroshi Okabe, Yoshiharu Sakai, Takashi Aoi

**Affiliations:** 1 Department of Reprogramming Science, Center for iPS Cell Research and Application, Kyoto University, Kyoto, Japan; 2 Department of Fundamental Cell Technology, Center for iPS Cell Research and Application, Kyoto University, Kyoto, Japan; 3 Department of Surgery, Graduate School of Medicine, Kyoto University, Kyoto, Japan; 4 Department of Gastroenterology Center, The Cancer Institute Hospital of Japanese Foundation for Cancer Research, Tokyo, Japan; 5 Department of iPS cell Applications, Graduate School of Medicine, Kobe University, Kobe, Japan; National Cancer Institute, United States of America

## Abstract

Cancer stem cells (CSCs) are considered to be responsible for the dismal prognosis of cancer patients. However, little is known about the molecular mechanisms underlying the acquisition and maintenance of CSC properties in cancer cells because of their rarity in clinical samples. We herein induced CSC properties in cancer cells using defined factors. We retrovirally introduced a set of defined factors (*OCT3/4*, *SOX2* and *KLF4*) into human colon cancer cells, followed by culture with conventional serum-containing medium, not human embryonic stem cell medium. We then evaluated the CSC properties in the cells. The colon cancer cells transduced with the three factors showed significantly enhanced CSC properties in terms of the marker gene expression, sphere formation, chemoresistance and tumorigenicity. We designated the cells with CSC properties induced by the factors, a subset of the transduced cells, as induced CSCs (iCSCs). Moreover, we established a novel technology to isolate and collect the iCSCs based on the differences in the degree of the dye-effluxing activity enhancement. The xenografts derived from our iCSCs were not teratomas. Notably, in contrast to the tumors from the parental cancer cells, the iCSC-based tumors mimicked actual human colon cancer tissues in terms of their immunohistological findings, which showed colonic lineage differentiation. In addition, we confirmed that the phenotypes of our iCSCs were reproducible in serial transplantation experiments. By introducing defined factors, we generated iCSCs with lineage specificity directly from cancer cells, not *via* an induced pluripotent stem cell state. The novel method enables us to obtain abundant materials of CSCs that not only have enhanced tumorigenicity, but also the ability to differentiate to recapitulate a specific type of cancer tissues. Our method can be of great value to fully understand CSCs and develop new therapies targeting CSCs.

## Introduction

Cancer stem cells (CSCs) have been suggested to be responsible for the poor prognosis of patients with various cancers due to their characteristics and behavior, such as higher rates of therapeutic resistance and recurrence [1]–[3]. Therefore, CSCs are regarded as a potential therapeutic target. To establish new treatments targeting CSCs, it is important to elucidate the molecular mechanisms underlying the acquisition of stemness in CSCs. However, these are still unclear, because CSCs are a rare population of cells in cancer tissue, and the rarity of the CSCs makes it difficult to identify and collect them.

Thus generating CSCs *in vitro* from cancer cells and investigating their characteristics is considered to be a useful method for overcoming this problem. Several studies [4]–[6] reported that cells with some CSC properties such as enhanced tumorigenicity were inducible. However they did not refer to whether the cells have differentiation ability to recapitulate specific types of cancer tissues. Therefore, it is still unclear whether it is possible to generate CSCs that precisely correspond to primary cancer stem cells.

With regard to acquisition of stemness, in the generation of induced pluripotent stem cells (iPSCs), it was found that the ectopic expression of only three or four transcription factors (*OCT3/4*, *SOX2* and *KLF4* with or without *C-MYC*) can induce embryonic stem cell properties in somatic cells when ESC culture conditions are used [7], [8]. These genes can also directly induce neural stem cell (NSC) properties in somatic cells using neuro-sphere culture conditions [9]. Thus, these genes have the ability to induce various types of stemness in somatic cells, depending on the culture conditions. Therefore, we hypothesized that these genes can also induce CSC properties in cancer cells.

In the current study, we transduced *OCT3/4*, *SOX2* and *KLF4* into human colon cancer cells under the parental cell culture conditions and analyzed the transduced cells in terms of their CSC properties *in vitro* and *in vivo*. Consequently, the set of three genes could induce CSC properties in a subset of colon cancer cells, and we were able to collect the cells with induced CSC properties based on their difference in dye-efflux activity. The collected cells showed colonic lineage specificity *in vitro* and *in vivo*. The induced CSCs generated using this method can help to investigate the molecular mechanisms underlying the acquisition and maintenance of CSC properties in cancer cells and to develop CSC-targeting therapy.

## Materials and Methods

### Cell lines and Cell Culture

Human colorectal cancer cell lines (SW480 and DLD-1) were supplied from the Cell Resource Center for Biomedical Research, Tohoku University. The cells were cultured in Dulbecco's modified Eagle's medium (DMEM) (Nacalai Tesque, Kyoto, Japan) containing 10% fetal bovine serum (FBS) (Life Technologies, Carlsbad CA, USA) and penicillin (100 Units/ml) and streptomycin (100 µg/ml) (Life Technologies), and used at early passage for the experiments.

### RNA isolation and quantitative reverse-transcriptase polymerase chain reaction

Total RNA was isolated using RNeasy Plus Mini Kit (QIAGEN, Hilden, Germany), according to the manufacturer's instructions. A 1 µg aliquot of total RNA was reverse transcribed by using a Transcriptor High Fidelity cDNA Synthesis Kit (Roche, Basel, Switzerland), according to the manufacturer's protocols. Quantitative reverse-transcriptase polymerase chain reaction (qRT-PCR) was performed with the FastStart Universal SYBR Green Master Mix (Roche) and was analyzed with the Step-One real-time PCR system (Life Technologies). The primer sequences are shown in Table S1.

### Flow Cytometry

A single cell suspension from cultured cells was immunostained with a mouse anti-human CD133 antibody (Clone: AC133, Miltenyi Biotec, Auburn CA, USA), mouse anti-human CD44 antibody (Clone: G44-26, Becton, Dickinson and Company [BD], Franklin Lakes NJ, USA) or mouse anti-human ABCG2 antibody (Clone: 5D3, BioLegend, San Diego CA, USA). After being washed, the cells were re-suspended with PBS containing 2% FBS, and dead cells were labeled by 2 µg/ml propidium iodide (PI, Sigma-Aldrich, St. Louis MO, USA). Single cell suspensions from paraformaldehyde-fixed cells were permeabilized with 0.2% Triton-X (Sigma), and were immunostained with a mouse anti-human CD26 antibody (Clone: M-A261, BD) and rabbit anti-human LGR5 (Clone: EPR3065Y, Abcam, Cambridge MA, USA). In all of the flow cytometry (FCM) experiments, isotype antibodies corresponding to each antibody were used as controls. The samples were analyzed by a FACS Aria II instrument (BD).

### Western blotting

The cells were lysed with the M-PER Mammalian Protein Extraction Reagent (Thermo Fisher Scientific, Rockford IL, USA). The cell lysates were subjected to SDS-polyacrylamide gel electrophoresis (SDS-PAGE). After the electrophoretic transfer of the proteins, immunoblotting with a mouse anti-human ALDH antibody (Clone: 44/ALDH, BD), followed by a horseradish peroxidase-conjugated secondary antibody, was performed. In order to determine the chemiluminescence, the LAS 4000 system (Fuji film, Tokyo, Japan) was used.

### Cell Cycle Analysis

The cells were fixed with 70% ethanol in PBS at 4°C overnight. The cells were treated with ribonuclease to digest RNA and stained with 50 µg/ml of PI. The cells were analyzed by flow cytometry (FACS Aria II).

### 5-FU-chemoresistance analysis

The cell viability after 5-fluorouracil (5-FU, KYOWA KIRIN, Tokyo, Japan) exposure was measured by the WST-8 colorimetric assay (Cell Counting Kit-8, Dojindo, Kumamoto, Japan). A total of 5×10^3^ cells were seeded in 96-well plates on day 10, and the medium was replaced with DMEM containing 1 or 50 µg/ml of 5-FU 24 hr after seeding. After incubation for 48 hr, the absorbance at 450 nm was measured using a microtiter plate reader. The cell viability was calculated as the ratio of absorbance values for the same sample incubated in DMEM without 5-FU for 48 hr.

### Sphere Formation Assay

The cells were transferred to Ultra Low Attachment plates (Corning Incorporated, Corning, New York, USA) in serum-free DMEM containing 10 ng/ml bFGF (Wako, Osaka, Japan), 10 µg/ml human insulin (CSTI, Miyagi, Japan), 100 µg/ml human transferrin (Roche) and 100 µg/ml BSA (Nacalai Tesque), and incubated at 37°C in a 5% CO_2_ incubator for 10 days.

### Dye Efflux Activity Analysis

A dye efflux activity analysis was performed according to previously described methods [10]–[12] with some modifications. The cells were harvested in DMEM containing 2% FBS and 1 mM HEPES (Sigma-Aldrich). The cells were incubated in DMEM containing 2% FBS and 1 mM HEPES with Hoechst33342 (Life Technologies) at 5 µg/ml with or without the co-administration of verapamil (Sigma-Aldrich) at 50 or 250 µM for 90 minutes at 37°C, and were gently inverted every 30 minutes. After incubation, the cells were re-suspended in PBS containing 2% FBS and 1 mM HEPES (Sigma-Aldrich). The cells were counterstained with 2 µg/ml PI to label dead cells, and were passed through a 35 µm mesh filter, keeping them on ice for the flow cytometry and sorting. Cells were analyzed and sorted by a FACS Aria II. The Hoechst dye was excited with a UV laser (355 nm), and the fluorescence was measured with both a 670/50 filter (Hoechst Red) and a 450/50 filter (Hoechst Blue).

### In Vivo Tumorigenicity Study

A total of 1×10^6^, 3×10^5^ or 1×10^5^ cells in 100 µl of serum-free PBS were injected subcutaneously into both dorsal flanks of an immunodeficient nude mouse (KSN/Slc mouse, SLC, Shizuoka, Japan). The tumor volume was calculated by the formula 0.5×L×W^2^ (L: length, W: width). The experiments were reviewed and approved by the Animal Ethics and Research Committee, Kyoto University (Permit Number: 11537, 12237, 13217), and conducted in accordance with institutional guidelines. All efforts were made to minimize suffering.

### Serial Transplantation

For the serial transplantation experiments, sorted cells were cultured for nine-16 days (first culture) after sorting, then a total of 3×10^6^ cells were subcutaneously injected into immunodeficient nude mice. The tumors (first tumors) were excised when they reached a diameter of more than 10 mm and were pathologically analyzed. The excised tumors were also enzymatically dissociated into single-cell suspensions by a gentleMACS Dissociator and Human Tumor Dissociation Kit (Miltenyi Biotec), according to the manufacturer's protocols. The dissociated cells were subcutaneously injected into nude mice after being cultured for six to ten days (second culture), and were analyzed by FCM based on their dye efflux activity on days six to twelve after the dissociation. The procedure was repeated three times until the fourth cultured cells from the dissociation of the third set of tumors were obtained.

### Immunohistochemistry

Formalin-fixed, paraffin-embedded sections derived from the xenografts were stained with anti-human cytokeratin 20 (CK20) mouse monoclonal antibody (Clone: Ks20.8, dilution 1∶25, Dako, Glostrup, Denmark), anti-human cytokeratin 7 (CK7) rabbit monoclonal antibody (Clone: SP52, concentration; 0.536 µg/ml, Roche) or anti-human caudal type homeobox 2 (CDX2) mouse monoclonal antibody (Clone: AMT28, dilution 1∶500, Leica Biosystems, Wetzlar, Germany) by the avidin-biotin immunoperoxidase method. Microwave antigen retrieval was performed. For immunocytochemistry, the cultured cells, which were fixed with 4% paraformaldehyde, were stained with anti-CK20 antibody (Clone: Ks20.8, dilution 1∶100) or anti-CDX2 antibody (Clone: CDX2-88, prediluted, Abcam), and counterstained with Hoechst33342 (Life Technologies) to identify all nuclei.

## Results

### Transduction of *OCT3/4*, *SOX2* and *KLF4* into a colon cancer cell line

We transduced *OCT3/4, SOX2, KLF4*, or a mixture of the three (hereafter, OSK) into the SW480 human colon cancer cell line using retrovirus vectors [7] (see Methods S1), and also a Mock vector (empty vector) was used as a control. These cells were then termed O-SW480, S-SW480, K-SW480, OSK-SW480 and M-SW480, respectively. The parental cells were also termed Wt-SW480. In this study, the cells were cultured in DMEM containing 10%FBS and evaluated on day 10 after transduction (Fig. S1A). We confirmed retroviral transduction and mRNA expression of transduced genes (Fig. S1B and S1C). In the M-SW480 cells, *KLF4* was endogenously expressed, while *OCT3/4* and *SOX2* were not detected. Distinguishable morphological changes were seen in each of the lines that were attributed to their transduced gene(s) (Fig. S1D).

### Expression of previously-reported markers related to colon CSCs and intestinal stem cells in transduced-SW480 cells

**Figure 1:**
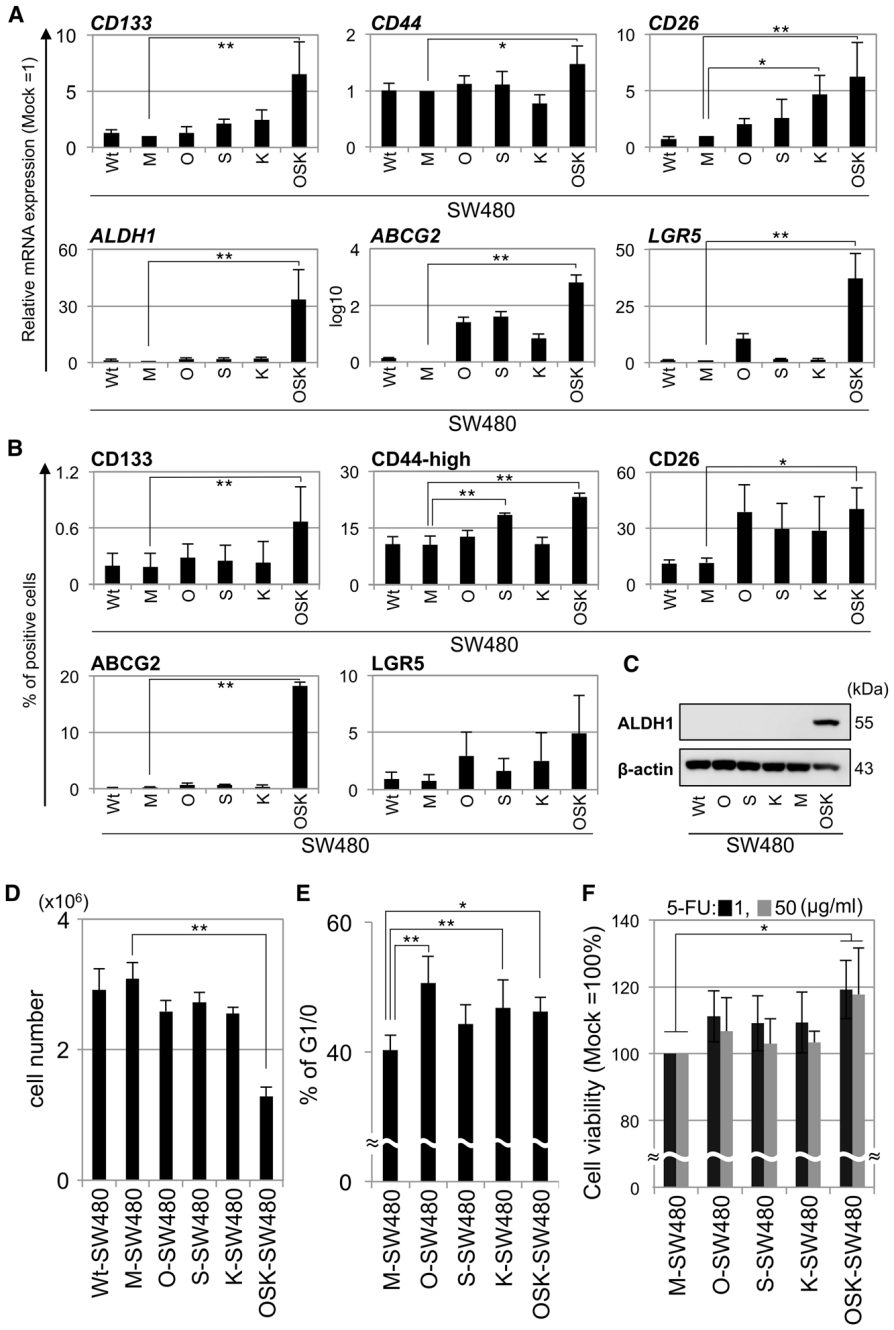
The transduction of OSK induced CSC properties in SW480 cells *in vitro*. (A) qRT-PCR of previously reported markers related to colon CSCs and intestinal stem cells in the transduced SW480 cells. All the markers were upregulated in the OSK-SW480 cells. The mRNA expression levels were normalized to those of *GAPDH*. The relative expression levels compared to those of M-SW480 are shown. (B) The protein expression levels of CSC markers in the transduced SW480 cells as determined by a flow cytometric (FCM) analysis. The data from three independent experiments demonstrated that the numbers of CD133-, CD44-high-, CD26- and ABCG2-positive cells were significantly increased in the OSK-SW480 cells. The representative dot plots of each marker are shown in Figure S2. (C) The protein expression of ALDH1 in the transduced SW480 cells determined by a Western blot analysis. The panel shows representative data from three independent experiments. (D) The cell proliferation *in vitro*. A total of 3×10^5^ cells were plated on six-well plates on day seven and were counted on day 11. The number of OSK-SW480 cells was lower than that of M-SW480 cells (n = 3). (E) The cells in the G1/0 phase were detected by a FCM analysis on day 11. The percentage of cells in the G1/0 phase significantly increased in the O-, K- and OSK-SW480 cells (n = 3). (F) 5-FU-chemoresitance analysis. The viability of OSK-SW480 cells in the presence of 5-FU was significantly higher than that of M-SW480 cells at both the 1 and 50 µg/ml concentrations of 5-FU (n = 3). The viability of the M-SW480 cells at each concentration was set to 100%. The error bars indicate the standard deviation: SD. *P<0.05, **P<0.01, Dunnett's test.

To assess the stem cell status of the transduced cells, we evaluated the expression levels of previously-reported candidate marker genes, albeit controversy [13], [14], of colon CSCs and intestinal stem cells, such as *CD133* [15], [16], *CD44* [17], [18], *CD26* [19], *ALDH1* [20], *ABCG2* [21] and *LGR5* [22] by qRT-PCR (Fig. 1A). Among the cell lines, only the OSK-SW480 cells had significantly increased mRNA expression levels of all the genes compared to M-SW480 cells (n = 3).

Next, we evaluated the protein expression levels of the markers (Fig. 1B, 1C and S2). The FCM analysis showed that the OSK-SW480 cells had significantly increased protein expression levels of CD133, CD26 and ABCG2. Most of the transduced cells expressed CD44, but the number of cells expressing a higher level of CD44 was increased around two-fold in OSK-SW480 cells compared to M-SW480 cells (n = 3) (Figs. 1B and S2). The OSK-SW480 cells showed a tendency to have an increased expression level of LGR5, but this was not statistically significant (Figs. 1B and S2) (n = 3). A western blot analysis showed that the expression level of ALDH1 was increased only in the OSK-SW480 cells (Fig. 1C). These results suggested that OSK have the ability to evoke CSC signatures in a subset of SW480 cells.

### Proliferation and cell cycle in transduced SW480 cells

We next assessed growth of the cells *in vitro*. The number of OSK-SW480 cells at 96 hr after seeding 3×10^5^ cells was significantly lower than that of M-SW480 cells (p<0.01, n = 3) (Fig. 1D). The longer observation also showed consistent results (Fig. S3). To examine if the transduced genes affected the cell cycle, we performed a cell cycle analysis by FCM with DNA staining using propidium iodide (Fig. 1E). The percentage of cells in the G1/0 phase was about 10% higher in O-, K- and OSK-SW480 cultures than that of the M-SW480 cells (n = 3).

### Sensitivity to chemotherapeutic agents

To examine the anticancer drug sensitivity of the cells, we compared the survival rates of the cells after treatment with 1 or 50 µg/ml of 5-FU for 48 hr by the WST-8 colorimetric assay (Fig. 1F). In the OSK-SW480 cells, the survival rate was around 20% higher than that of the M-SW480 cells at both concentrations of 5-FU (n = 3). This result suggested that OSK-SW480 cells contained the cells acquired chemoresistance to 5-FU.

### Sphere formation assay

**Figure 2:**
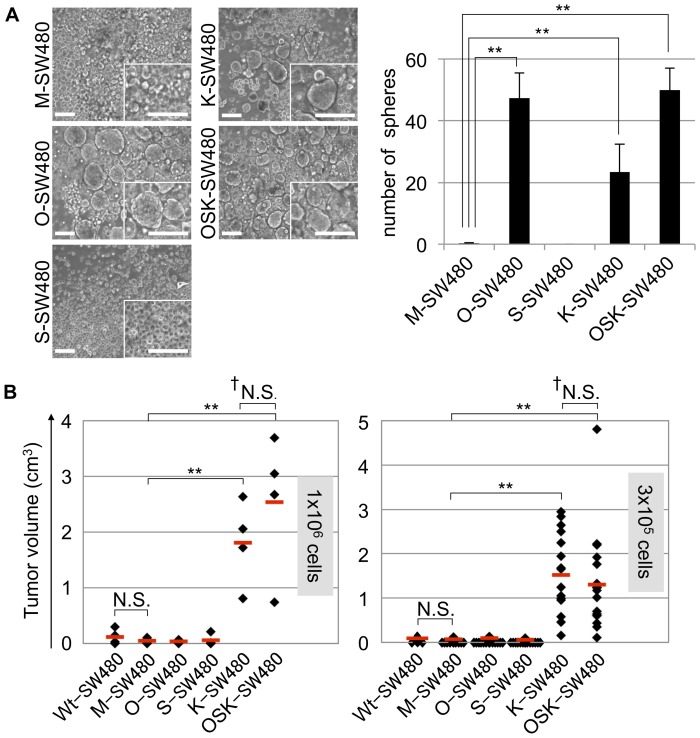
The sphere formation ability *in vitro* and tumorigenicity *in vivo*. (A) The spheroid formation assay. A total of 1×10^4^ cells were plated on low attachment dishes on day 10 and cultured with serum-free medium for 10 days. In the O-, K- and OSK-SW480 cultures, the number of spheroids was significantly increased compared to that of the M-SW480 cells. The error bars indicate the SD (n = 3). Scale bars: 100 µm. (B) The tumorigenicity of the cells after implantation in the subcutaneous regions of immunodeficient nude mice. A total of 1×10^6^ cells (left panel) or 3×10^5^ cells (right panel) were subcutaneously injected into both flanks of immunodeficient nude mice on day 10. The volume of the tumors derived from the K- and OSK-SW480 cells were obviously higher than those of the Wt-, M-, O- and S-SW480 cells for both numbers of injected cells. The red bars indicate the median tumor volume. **P<0.01, N.S.: not significant, Dunnett's test (except of †: U-test).

It has previously been reported that CSCs had a higher ability to form spheroids under culture in low attachment dishes and serum-free medium [2], [16]. To examine the sphere-forming ability of our transduced cells, we performed a sphere formation assay (Fig. 2A). In the M-SW480 cells, we hardly observed any spheroids. In contrast, in the O-, K- and OSK-SW480 cultures, we observed an obviously increased number of the spheroids (average: 47, 23 and 50, respectively, n = 3), indicating that *OCT3/4, KLF4* and OSK contributed to the spheroid formation in a subset of SW480 cells.

### Tumorigenicity *in vivo*

To examine the tumorigenicity of the transduced cells, we subcutaneously transplanted 1×10^6^ or 3×10^5^ cells into immunodeficient nude mice in the dorsal area on both sides, and then we measured the incidence and volume of tumors after four weeks for the 1×10^6^ cells and eight weeks for the 3×10^5^ cells after transplantation, respectively (Fig. 2B). A summary of the tumor incidence is shown in Table 1. The K- and OSK-SW480 cells generated tumors in 100% of the locations, whereas the other lines generated tumors in 75% and 25% of the cases for the cell number of 1×10^6^ and 3×10^5^ cells, respectively. We observed a significantly higher volume of tumors in the K- and OSK-SW480 cell-injected mice compared to those injected with other lines, at both cell amounts, namely 1×10^6^ and 3×10^5^ cells (Fig. 2B).

**Table 1:** Summary of tumor formation derived from transduced SW480 cells.

	Tumor formation	
	Injected cell number	
Cell name	1×10^6^	3×10^5^
Wt-SW480	75% (3/4)	25% (1/4)
M-SW480	75% (3/4)	25% (3/12)
O-SW480	75% (3/4)	25% (4/16)
S-SW480	75% (3/4)	25% (4/16)
K-SW480	100% (4/4)	100% (16/16)
OSK-SW480	100% (4/4)	100% (*17/16)

*In one case, we observed 2 tumors in one injected region.

Taken together, these data indicate that OSK sufficiently induced all of the previously reported CSC properties we examined *in vitro* and *in vivo*, whereas no single gene was sufficient to induce all of these properties. We designated the cells with the CSC properties in the OSK-SW480 cultures as induced CSCs (iCSCs).

### Efflux activity for Hoechst33342

In previous reports [2], [10], [12], [23], [24], FCM using Hoechst33342 labeling with verapamil (VM), which is an ATP-Binding Cassette (ABC) transporter inhibitor, was an effective way to enrich various types of stem-like cells, including cancer cells. In this assay, it was considered that the stem-like cells (so-called Side Population cells) were not labeled by Hoechst33342 without VM, but were labeled by Hoechst33342 with the administration of VM.

**Figure 3:**
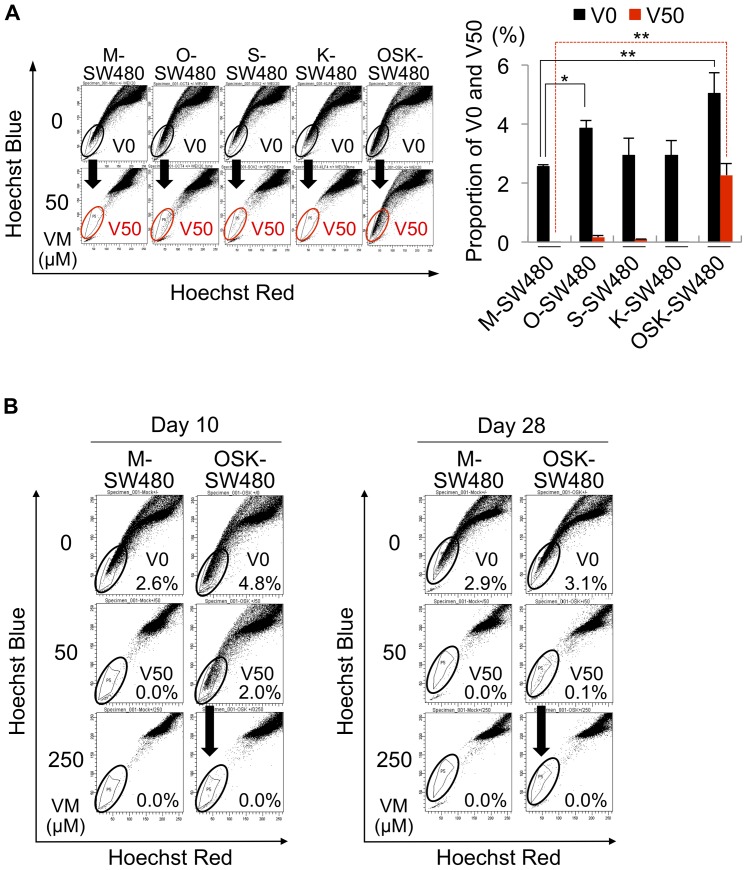
Efflux activity for Hoechst33342. (A) The OSK-SW480 cells included a population of cells unlabeled by 5 µg/ml of Hoechst33342 with the co-administration of 50 µM of verapamil (VM). We designated the cells unlabeled by Hoechst33342 without VM and with 50 µM of VM as V0-cells and V50-cells, respectively. The left panel shows representative dot plots of labeled and unlabeled cells at day 10 after transduction. The right panel shows the results of three independent experiments. The V0 subpopulation was increased in the O- and OSK-SW480 cells. The V50-cells were obviously seen in the OSK-SW480 cultures. The error bars indicate the SD (n = 3). *P<0.05, **P<0.01, Dunnett's test. (B) Dye efflux activity at day 10 (left panel) and day 28 (right panel) after transduction. The V50-cells disappeared under the treatment with the co-administration of 250 µM of VM even in the OSK-SW480 cells. The proportion of the OSK-V50 cells decreased with time.

We confirmed the existence of 5 µg/ml of Hoechst33342-effluxing cells, which disappeared with the co-administration of 50 µM of VM in the M-SW480 culture (Fig. 3A). We set a gate in which the population was included, and termed the cells in the gate in the absence of VM as “V0-cells” (Fig. 3A, left panel), followed by an analysis of other lines. The number of V0-cells significantly increased in the O- and OSK-SW480 cultures (3.9% and 5.0%, respectively) compared to the M-SW480 culture (2.6%) (Fig. 3A, right panel). Notably, unique cells, which were unlabeled by Hoechst33342 even with the presence of 50 µM of VM, were obvious in the OSK-SW480 cultures. We termed these cells as “V50-cells” (Fig. 3A, left panel). Treatment with 250 µM of VM resulted in the disappearance of the cells within the gate, indicating that the V50-cells were sensitive to 250 µM of VM (Fig. 3B, left panel). Taken together, these data indicated that we obtained a new collectable cell population: V50-cells with a highly potent dye-efflux activity induced in the OSK-SW480 cultures.

### Confirmation with another colon cancer cell line

To confirm the current results, we examined another colon cancer cell line, DLD-1, in some experiments, including evaluations of the cell growth rate *in vitro*, tumorigenicity *in vivo* and the Hoechst33342 effluxing properties (Fig. S4). In the DLD-1 cells, the growth rate of the OSK-DLD-1 cells was lower than that of the Wt- (parental) and Mock-DLD-1 cells (p<0.01, n = 3) (Fig. S4A). The tumorigenicity of 1×10^5^ cells was higher in OSK-DLD-1 cells compared to Wt- and Mock-DLD-1 cells (Fig. S4B, Table S2). V50-cells were also seen in the OSK-DLD-1, but not in the Mock-DLD-1, cultures (Fig. S4C).

### Collecting the iCSCs from OSK-SW480

To examine whether the CSC properties induced in OSK-SW480 cultures were attributable to V50-cells, we sorted and analyzed the V50-cells and non-V50-cells in the presence of 50 µM of VM in OSK-SW480 cells, and V0-cells and non-V0-cells in the absence of VM and non-V50-cells in the presence of 50 µM of VM in the M-SW480 cultures. These cells were termed OSK-V50, OSK-nonV50, M-V0, M-nonV0 and M-nonV50, respectively.

**Figure 4:**
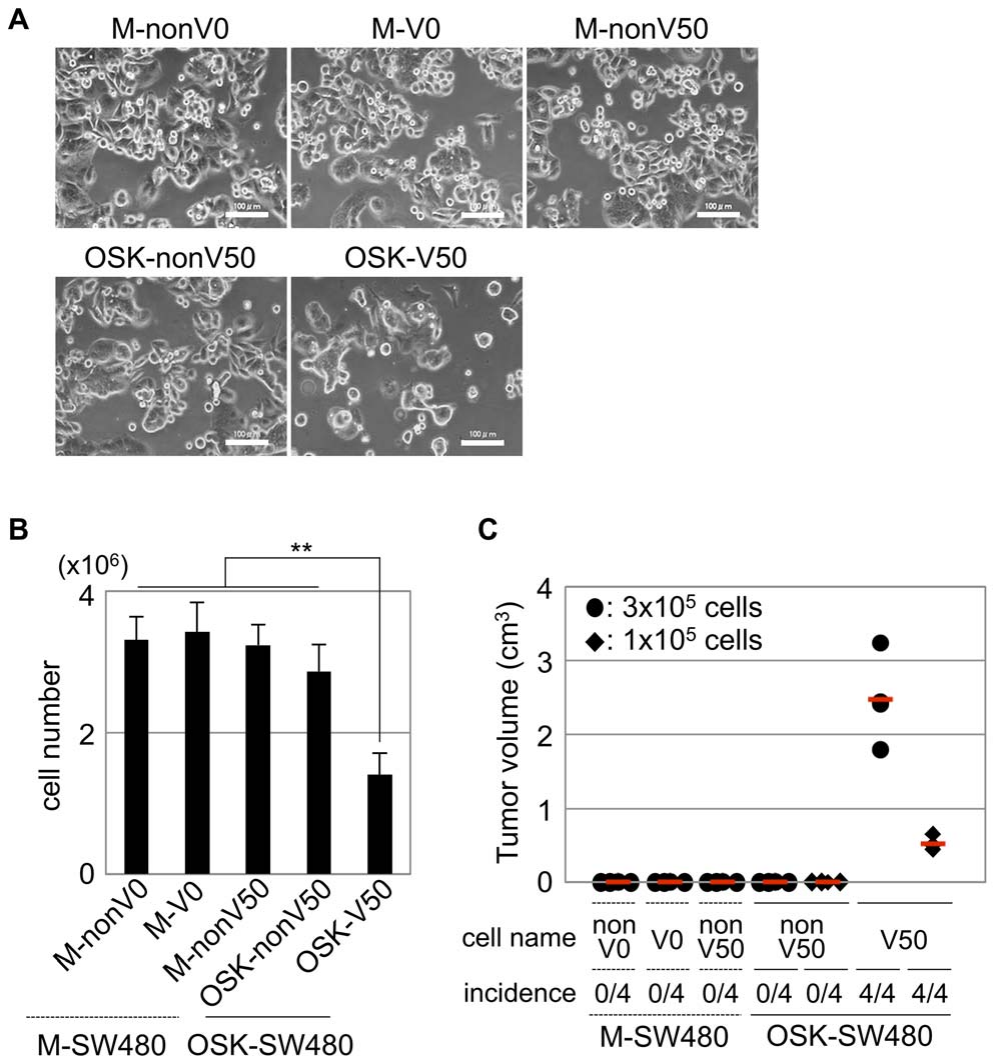
Characterization of the V50-cells in OSK-SW480 cells after FACS. The V50-cells in the OSK-SW480 (OSK-V50) cells were sorted by FACS. Non-V0-, V0- and non-V50-cells in the M-SW480 cells (M-nonV0, M-V0 and M-nonV50, respectively) and non-V50-cells in the OSK-SW480 cells (OSK-nonV50) were also sorted and used as controls. These cells were all subsequently cultured. (A) The morphologies of the cells cultured for 10 days after sorting. The morphology of the OSK-V50 cells was similar to that distinctively observed in OSK-SW480 cells (Fig. S1D, lined circle). In contrast, the morphology of OSK-nonV50 cells was similar to that of M-V0, M-nonV0 and M-nonV50 cells. Scale bars: 100 µm. (B) The cell proliferation *in vitro*. A total of 3×10^5^ cells cultured for 14 to 18 days after sorting were seeded and counted 96 hr later. The number of cells was significantly lower in the OSK-V50 cells than that in all the other lines. The error bars indicate the SD (n = 3). **P<0.01, Scheff's test. (C) Tumorigenicity of the cells in immunodeficient mice. A total of 3×10^5^ or 1×10^5^ cells were subcutaneously injected into immunodeficient nude mice on day 18 after sorting. The tumor volume and incidence were measured eight weeks after injection. Only the OSK-V50 cells generated obvious tumors for both the injected cell numbers, whereas no tumors were obtained from the M-nonV0, M-V0, M-nonV50 and OSK-nonV50 cells. The incidence of tumor formation by OSK-V50 cells was 4/4 for both injected cell numbers. The red bars indicate the median tumor volume.

After sorting by a fluorescence-activated cell sorter (FACS) on day 10, all the lines were subsequently cultured for 10 days in DMEM containing 10% FBS. The OSK-V50 cells exhibited morphology similar to that distinctively observed in the OSK-SW480 cells on day 10 (Fig. 4A, Fig. S1D). In contrast, the OSK-nonV50 cells exhibited morphology similar to that of the M-V0, M-nonV0 and M-nonV50 cells (Fig. 4A). The cell growth rate of the OSK-V50 cells was significantly lower than that of the other lines (p<0.01, n = 3) (Fig. 4B), resulting in decreased proportion (∼0.1%) of the V-50 cells at 28 days after transduction under the current culture condition (Fig. 3B, right panel).

The tumorigenicity of the OSK-V50 cells in the immunodeficient mice was obviously higher in terms of the size and incidence of tumors than that of the other cell lines, including OSK-nonV50 cells (Fig. 4C).

Taken together, these data indicate that the OSK-V50 cells exhibited CSC properties, but that the OSK-nonV50 cells did not, indicating that the CSC properties induced in OSK-SW480 cells were attributable to the V50-cell population.

### Colonic lineage differentiation of OSK-V50 cells *in vitro*

**Figure 5:**
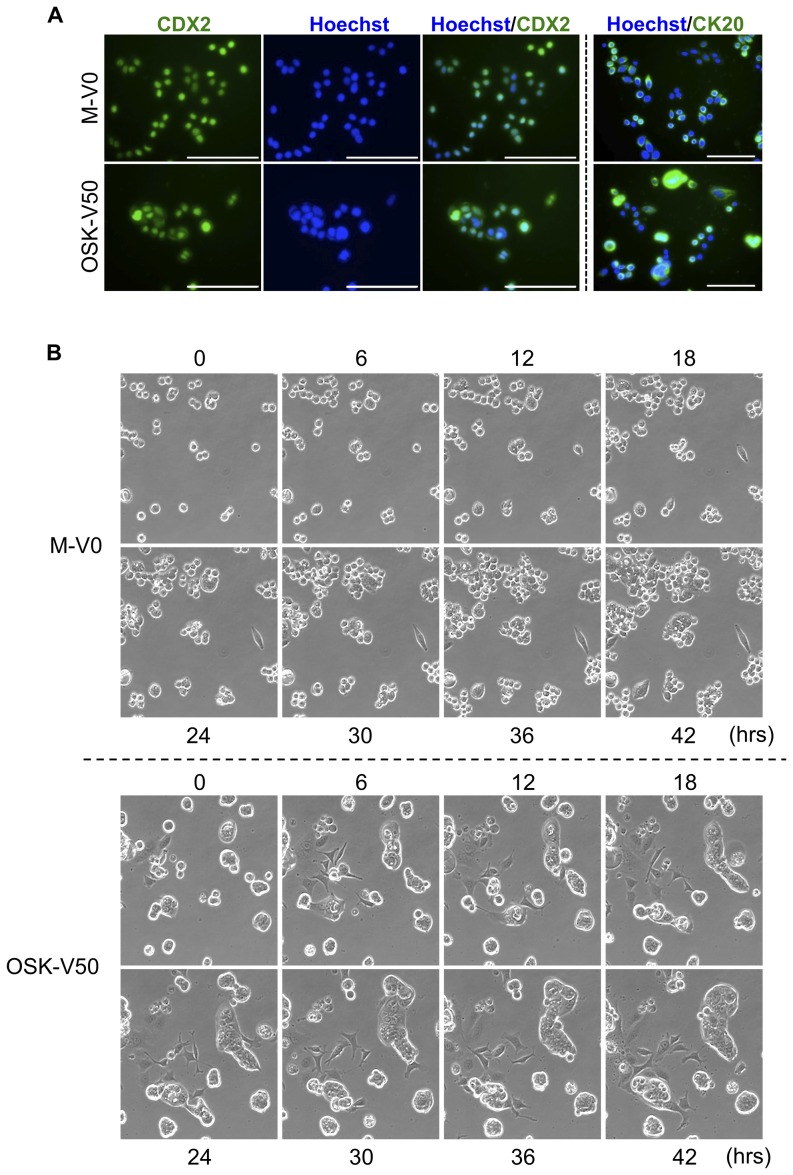
Colonic lineage specificity and potency to produce phenotypical diversity in OSK-V50 cells. (A) Immunocytochemistry for CDX2 and CK20 protein. OSK-V50 cells were positive for CDX2 and CK20, as well as M-V0 cells. The cells were counterstained with Hoechst33342. Scale bars: 100 µm. (B) The phenotypical diversity in morphology and mobility of derivatives of OSK-V50 cells. Photographs are time-lapse images of M-V0 and OSK-V50 cells. The sorted cells were subsequently cultured for five days and then observed every six hours for 42 hr. Time-lapse imaging revealed that there was a higher diversity in both the morphology and mobility in OSK-V50 cells (lower panel) compared to M-V0 cells (upper panel). Original magnification, ×20. Movies of M-V0 and OSK-V50 cells are shown in Video S1 and Video S2, respectively.

OSK-V50 cells as well as M-V0 cells were positive for CDX2, a master regulator of intestinal epithelial differentiation [25], and CK20, a colonic differentiation marker [26], by immunostaining (Fig. 5A). Thus OSK-V50 cells maintained colonic lineage phenotype and differentiation ability *in vitro* (Fig. 5A).

### Phenotypical diversity in derivatives of OSK-V50

Generating heterogeneity in cancer tissues is one of the notable CSC properties [1]–[3]. To examine this property in our isolated cells, we subsequently analyzed 23 days after sorting (Fig. S5). The OSK-V50 cells, in contrast to all the other cells, could produce V50-cells and as well as diverse different subsets of cells in terms of the degree of Hoechst-effluxing function (Fig. S5).

We next performed *in vitro* time-lapse imaging of M-V0 and OSK-V50 cells for 42 hr (Fig. 5B, Methods S1). The M-V0 cells consisted of small round- and spindle-shaped cells, and formed colonies consisting of cells with clear edges. The OSK-V50 cells consisted of cells with various morphologies, such as polygonal-, round-, cuboidal-, spindle- and flat-shaped cells, which altered their morphologies with time, and formed flat-mounted colonies consisting of cells with unclear edges. Furthermore, the OSK-V50 cells exhibited higher cell mobility than the M-V0 cells (Fig. 5B, Videos S1 and S2). These results suggest that OSK-V50 cells can produce a higher diversity in terms of morphology and mobility compared to M-V0 cells.

### Colonic lineage differentiation of OSK-V50 cells *in vivo*

**Figure 6:**
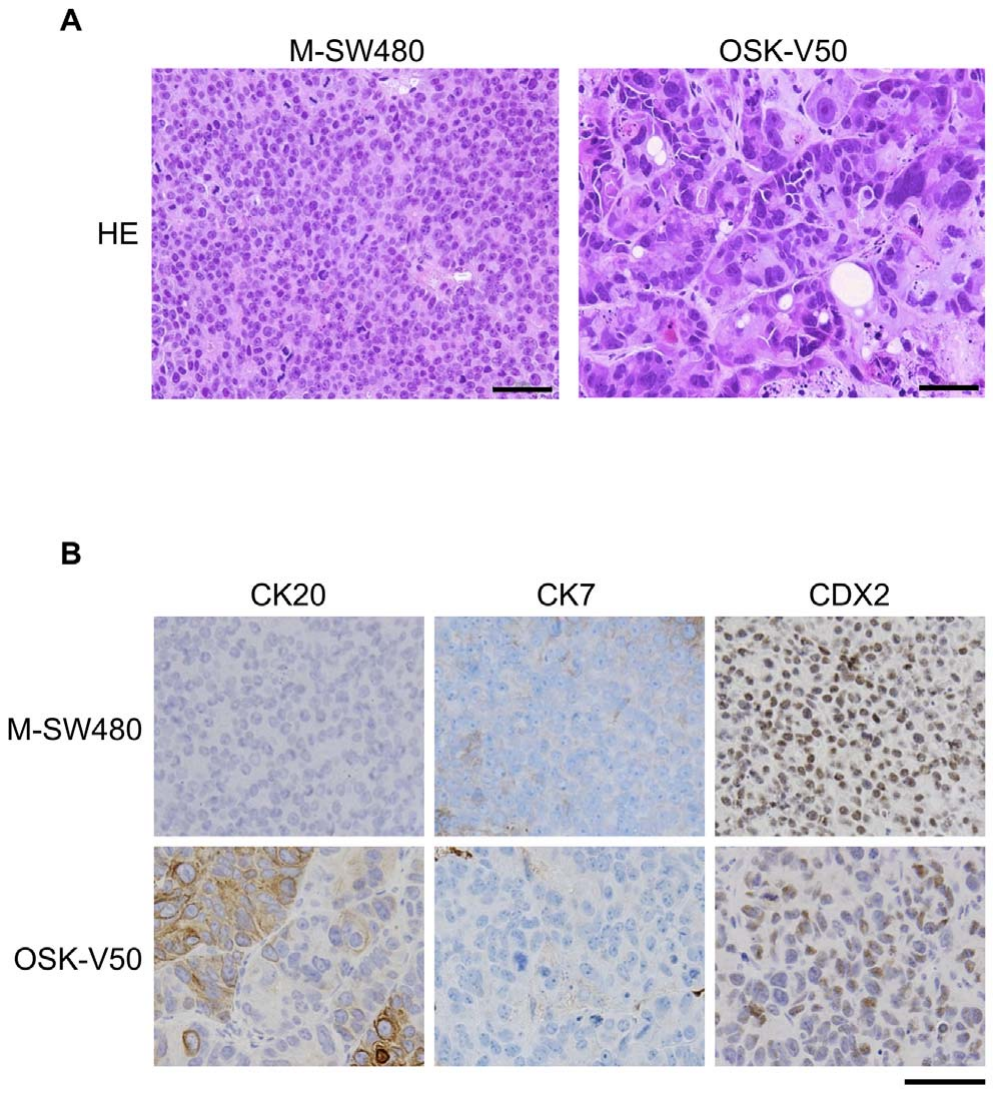
The histology of the xenografts derived from M-SW480 and OSK-V50 cells. (A) Hematoxylin and eosin staining (HE). The tumors derived from M-SW480 cells predominantly consisted of homogeneous cells. On the other hand, the tumors derived from OSK-V50 cells showed glandular structures. (B) Immunohistochemical analysis. The tumors derived from M-SW480 cells were negative for CK20 and CK7. The tumors derived from OSK-V50 cells were partially positive for CK20 and negative for CK7. Tumors derived from both M-SW480 and OSK-V50 cells were positive for CDX2, but CDX2-negative cells were obviously seen in the tumors of OSK-V50 cells. Scale bars: 50 µm.

We next histologically assessed the tumors derived from the M-SW480 and OSK-V50 cells in immunodeficient mice (Fig. 6). The tumors derived from the M-SW480 cells predominantly consisted of the homogeneous and monotonous expansion of uniform dysplastic cells. In contrast, in the tumors derived from OSK-V50 cells, we observed cell diversity and glandular structures, which are often present in human colorectal cancer tissues (Fig. 6A).

In addition, we performed an immunohistochemical analysis (Fig. 6B). Typical human colon cancer tissues are positive for CK20 and CDX2, and negative for CK7 [27], [28]. The tumors derived from the M-SW480 cells were negative for CK20 and CK7. In contrast, the tumors derived from OSK-V50 cells were partially positive for CK20 and negative for CK7, which was consistent staining pattern with actual human colon cancer tissues [29], [30]. Together OSK-V50 cells showed colonic lineage phenotype and differentiation ability *in vivo*. Furthermore, the tumors generated from M-SW480 cells were positive for CDX2, whose expression level is reduced in the later stages of human colorectal cancer and invasive cancer indicated by an immunohistochemical study [31]. The tumors derived from OSK-V50 cells were also positive for CDX2, whereas these tumors contained more CDX2-negative cells than the tumors derived from M-SW480.

These data demonstrated that the tumors of OSK-V50 cells represented a similar in histological and immunohistochemical characteristics to actual human colon cancer tissues.

### Self-renewal capacity of the OSK-V50 cells *in vivo*

**Figure 7:**
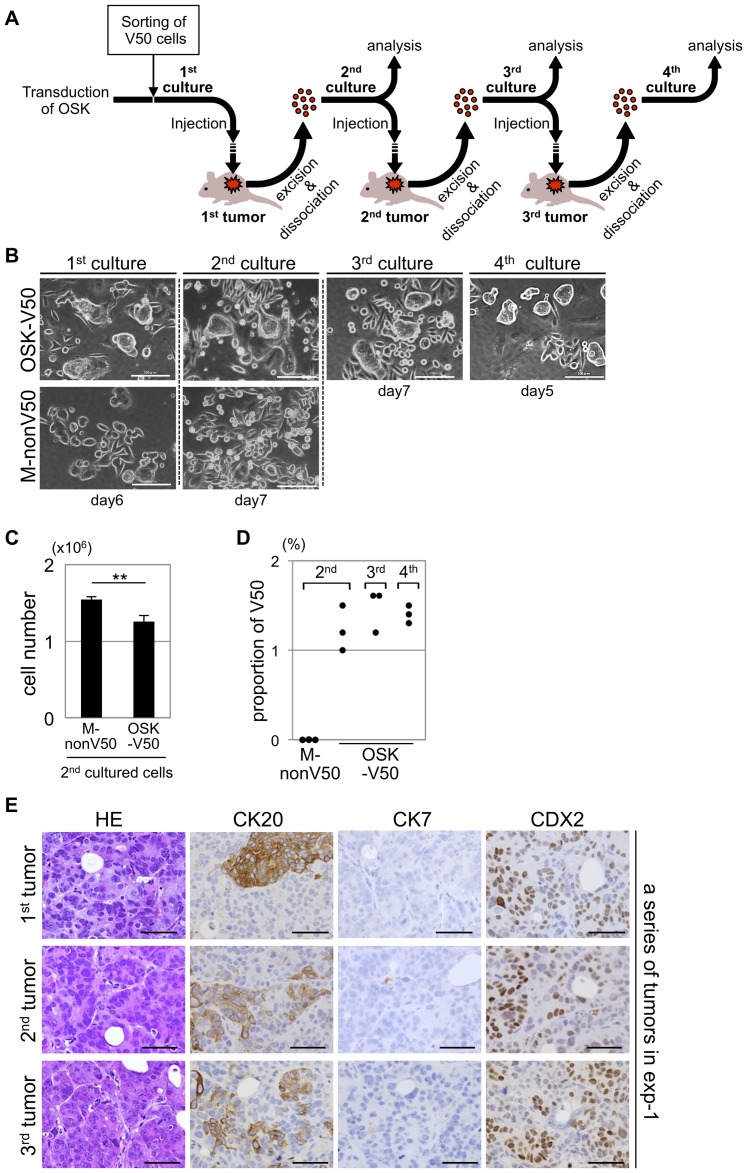
The self-renewal capacity of the OSK-V50 cells *in vivo*. (A) A schematic representation of serial transplantation experiments. (B) The morphological features of a series of cultured cells in the serial transplantation experiments. The day shown under the panel indicates the culture period after sorting for the first cultured cells and after dissociation from tumors for the second, third and fourth cultured cells. In the case of OSK-V50 cells, the morphological features in each culture were the same over three passages *in vivo*, and the features were similar to those shown in Figures 4A and 5B. Scale bars: 100 µm. (C) The cell proliferation of the second cultured cells *in vitro*. A total of 2×10^5^ cells cultured for four to six days after dissociation were seeded and counted 72 hr later. In the second cultured cells, the number of OSK-V50 cells was significantly lower than that of M-nonV50 cells (n = 3). **P<0.01, t-test. (D) The proportion of V50 cells in the second, third and fourth cultured cells. The panel shows the proportion of V50 cells in each culture from three independent experiments. The V50 cells were maintained over three passages *in vivo*, whereas V50 cells were not observed in the second M-nonV50 cultured cells. The representative dot plots obtained by FCM are shown in Figure S6. (E) The pathological findings of xenograft tumors derived from OSK-V50 cells. The panel shows the histological findings of a series of tumors obtained from serial transplantation experiments. The results of the other series of tumors are shown in Figure S7. The OSK-V50 cells serially showed the same pathological findings as those described in Figures 6A and 6B; there was cell diversity and glandular structures observed by HE staining, and CK20- and CDX2-positive, and CK7-negative findings for the immunostaining. exp: experiment. Scale bars: 50 µm.

To examine whether the OSK-V50 cells could self-renew *in vivo*, we next performed serial transplantation experiments (n = 3). A schematic representation of the serial transplantation experiments is shown in Figure 7A. In this experiments, a total of 3×10^6^ cultured OSK-V50 cells (first cultured cells) were subcutaneously injected into nude mice. The tumors derived from these first cultured cells were dissociated, and the dissociated cells were subsequently cultured (second culture) *in vitro*. The tumors and the dissociated cells were analyzed. We repeated the procedure three times until the fourth culture of cells. From the OSK-V50 cells, we could serially obtain tumors large enough for the following procedures. Whereas in the case of M-nonV50 cells which we used as control cells in this experiments, the transplantation of M-nonV50 cells at the same number as that used for the OSK-V50 cells led to the formation of only a small nodule. Therefore, we injected a total of 8–10×10^6^ cells from the first cultured M-nonV50 cells, and performed the serial transplantation experiments only once until the second culture of M-nonV50 cells.

The first, second, third and fourth serially cultured OSK-V50 cells showed the same morphological characteristics, which were similar to those shown in Figures 4A and 5B (Fig. 7B). The number of OSK-V50 cells 72 hr after seeding 2×10^5^ cells was significantly lower than that of M-nonV50 cells in the second culture (Fig. 7C) (p<0.01, n = 3). In the dye efflux activity analysis, the proportion of V50 cells was 1–1.6% in the second, third and fourth cultures of cells in case of the OSK-V50 cells, whereas no V50 cells were observed in the second culture of the M-nonV50 cells (Figs. 7D and S6). In addition, the first, second and third tumors serially derived from the OSK-V50 cells exhibited the same histological and immunohistochemical findings, which were similar to actual human colon cancer tissues, as shown in Figure 6 (Figs. 7E and S7). In contrast, the tumors derived from M-nonV50 cells showed the same features as those of the M-SW480 cells shown in Figure 6, not the same as the findings seen for OSK-V50 cells. These data indicated that the OSK-V50 cells showed self-renewal capacity in the serial *in vivo* transplantation studies.

### Characterization of the re-sorted V50 cells from tumors

We next collected V50 and non-V50 cells from the third cultured OSK-V50 cells by FACS sorting based on the dye-effluxing ability of the cells, and analyzed the cells (n = 3) (Fig. S8A). The number of the re-sorted V50 cells 96 hr after seeding 3×10^5^ cells was significantly lower than that of the re-sorted non-V50 cells (p<0.01, n = 3) (Fig. S8B). The re-sorted V50 cells exhibited the same morphological features as the OSK-V50 cells, as shown in Figures 4A and 5B, showing a higher diversity in their morphologies with time (Fig. S8C). A total of 3×10^6^ cells of the re-sorted V50 cells cultured for five to six days were subcutaneously injected into nude mice. The tumors derived from the re-sorted V50 cells showed the same pathological findings as the first, second and third tumors (Fig. S8D). The proportion of V50 cells in the cultured cells from tumors derived from the re-sorted V50 cells was 1.3–2.7% (Fig. S8E). These data support that the re-sorted V50 cells can also self-renew *in vivo*.

In summary, we generated the cells with CSC properties: iCSCs, by introducing *OCT3/4*, *SOX2* and *KLF4* in the SW480 colon cancer cell line, and were able to isolate an iCSCs-enriched cell population by using Hoechst33342 staining and VM treatment. The tumors derived from the iCSCs exhibited a more similar phenotype to *bona fide* colon cancer than did those from the parental SW480 cell line.

## Discussion

In this study, we found that a subset of colon cancer cells sufficiently acquired the colon CSC properties by introduction of OSK. It was previously reported that these transcription factors can convert somatic cells into iPSCs [7], [8] and neural stem cells depending on their culture conditions [9]. Therefore, in the current study, we considered that OSK directly induced a cell-fate conversion to a CSC state in a subset of SW480 cells under their primary culture conditions, not *via* an iPSCs state.

Our data showed that forced expression of OSK could directly generate colon CSCs from colon cancer cells. On the other hand, recent studies reported that “CSCs” could be generated from iPSCs in both mouse [4] and human studies [5]. In a mouse study, CSCs giving rise to “adenocarcinoma” could be generated from iPSCs derived from normal mouse embryonic fibroblasts. In a human study, iPSCs-derived CSCs, whose origin was human mammary epithelial cells, could form “multilineage tumors”. However, the iPSCs-derived CSCs in both cases were originally generated from “non-cancer cells”. It is generally considered that cancer initiation involves genetic changes [32]. The tumors from the iPSCs-derived CSCs in both reports were less likely to have the pathogenic genetic changes associated with “cancer”, therefore, it is still unclear whether the iPSCs-derived CSCs represent the CSC properties present in actual cancer cells which carry oncogenic genetic alterations. It should be noted that in this study, we succeeded in generating iCSCs from SW480 colon cancer cells, which have a pathogenic genome of “colon cancer”.

In the current study, we were able to collect the cells with induced CSC properties based on their difference in the dye-efflux activity. We originally focused on the differences in the degree of efflux activity, and succeeded in establishing a new method to distinguish V50-cells from V0-cells in the OSK-SW480 cells by changing the concentration of VM. By using this method, we clarified that the forced expression of OSK induced not only an increase in the frequency of V0-cells existing in the SW480 cultures, but also the emergence of V50-cells that gained more enhanced effluxing activity.

The efflux pump activity is an important property in CSCs [33], because the efflux pump eliminates metabolic products and toxic compounds. Therefore, V50-cells are considered to be better able to preserve their survival even in a hostile environment, such as following treatment with chemotherapy or metastatic regions, in comparison to V0-cells that enriched primary CSCs [23], [34]. CSCs are not uniform [2], [35], thus it is important to consider not only the frequency of CSCs, but also the differences in the degree of their stemness.

The present results indicated that the OSK-V50 cells have colonic differentiation potency *in vitro* and *in vivo*. In the immunohistological study, the tumors derived from OSK-V50 cells mimicked *bona fide* colon cancer tissue and keep their lineage as colon cancer [27], [28], [36]. In contrast, the tumors of M-SW480 cells did not. We confirmed that these phenotypes of our iCSCs were reproducible in serial transplantation experiments using xenograft models. In addition, CK20 is well known as a marker of differentiation in colorectal cancer [26], therefore, the expression of CK20 in the xenografts of OSK-V50 cells suggests that OSK induced the ability of the OSK-V50 cells to differentiate, leading to higher cell diversity *in vivo*. These findings were consistent with the principle of a hierarchy as advocated by the CSC concept. In terms of the clinical applications of these cells, such as the development of anti-colon CSC drugs, it is critical to develop tumors that recapitulate *bona fide* colon cancers. The previous reports did not focus on the histology of tumors derived from iCSCs in detail, such as the structure, phenotypic diversity (based on the differentiation capacity of the iCSCs) and the lineage of the original tissues [4], [5]. There exist “cancers of unknown origin”, but not “cancers of no origin”. Therefore, it is a significant point that the current method can allow for tumors similar to actual human colon cancer to be formed even in the subcutaneous region in mice.

In the current study, we could induce CSC properties in colon cancer cells by using an artificial system involving the forced expression of *OCT3/4*, *SOX2* and *KLF4*. Although these factors were individually reported to be correlated with the malignant behavior and poor prognosis in various cancers [37]–[42], it is unclear whether there are any cells that spontaneously overexpress *OCT3/4*, *SOX2* and *KLF4* in colon cancer tissues. Nevertheless, the important finding of this study was that the current colon iCSCs can form tumors similar to actual colon cancer, while the parental cell line could not. This finding implied that some kind of unknown OSK-downstream molecules might play a key role in our iCSCs, resulting in recapitulation of colon cancer tissues. Furthermore, if we identify the key molecules that are required for the development of actual colon CSCs, our iCSCs will help to establish CSC-targeting therapy by overcoming the sampling limitation of CSCs in clinical specimens. Therefore, a global analysis of the transcriptome, as well as carrying out proteomic and epigenomic studies could be useful to find such key molecules in future studies.

It could be a point of interest that only a small subset of transduced cells became iCSCs, and understanding the reason for this might help to better understand the generation of iCSCs. Although we did not evaluate the exact percentage of cells that expressed exogenous OCT3/4, SOX2 and KLF4 in the current study, there seemed to be a discrepancy between the percentage of OSK-V50 cells (∼2%) and the retroviral transduction efficiency (∼35% or more) of OSK inferred from Figure S1B, in which around half of the cells infected with the retrovirus mixture carrying one of three factors expressed both eGFP and DsRed. Therefore, the efficiency of the generation of the iCSCs might not simply reflect the transduction efficiency of OSK. It is still to be elucidated whether there are any mechanisms associated with suppressing the process of iCSC generation, like those seen for iPSC generation [43].

In addition, although we demonstrated that our iCSCs maintained their colon CSC nature *in vivo*, it is also still unclear whether the continuous presence of the exogenous OSK factors is needed for the maintenance of this colon CSC nature. To address this issue, it will be necessary to use a transient expression system for OSK in future studies.

In summary, we were able to generate colon iCSCs from an established colon cancer cell line by forced expression of OSK, and collect the iCSCs based on their difference in dye efflux activity. The iCSCs-derived cells and tissues were similar to actual human colon cancer tissue. By overcoming the quantitative limitations of primary human CSC samples and by dynamic observation of the CSC development, this method will enable us to elucidate the molecular mechanisms involved in the development and maintenance of CSCs, and will help to establish new therapies and diagnostic technology targeting CSCs.

## Supporting Information

Figure S1 Transduction of *OCT3/4, SOX2, KLF4* or a mixture of the three factors (OSK) in SW480 cells. (A) A schematic representation of this study. (B) The transduction efficiency in SW480 cells. Cells were retrovirally transduced with a mixture of three factors; eGFP, DsRed and Mock (empty vector). Almost all cells expressed at least one the transduced genes. Around half of the cells expressed both eGFP and DsRed. Scale bar: 100 µm. (C) qRT-PCR of *OCT3/4*, *SOX2* and *KLF4* in transduced SW480 cells using primers common for both endogenous and exogenous transcripts. The mRNA expression levels were normalized to those of *GAPDH*. The relative expression levels compared to those to human iPSCs are shown. All the transduced genes were obviously expressed. The error bars indicate the SD (n = 3). † : not detected. (D) The morphology of the transduced SW480 cell lines. The transduction of each individual factor led to distinct morphological changes (arrow, dotted circle or arrow head). Simultaneous transduction of the three factors also led to specific morphologic features (lined circle). Parental cells (Wt-SW480) and M-SW480 cells predominantly consisted of spindle-shaped cells and small round-shaped cells. In the O-SW480 culture, globular clusters consisting of cells with unclear edges appeared. In the S-SW480 cultures, the number of round-shaped cells was increased. In K-SW480 cultures, colonies consisting of cells with slightly unclear edges appeared. In the OSK-SW480 cultures, flat-mounted colonies consisting of cells with unclear edges appeared, and similar morphologies were also seen as were noted in the other cells. Scale bars: 100 µm.
(PDF)

Figure S2 The results of the flow cytometric (FCM) analysis of the CSC marker protein expression in transduced SW480 cells. The panel shows representative dot plots of the cells expressing CD133, CD44, CD26, ABCG2 and LGR5 in the transduced SW480 cells.
(PDF)

Figure S3 Cell proliferation of transduced SW480 cells *in vitro*. The cell number of transduced SW480 cells was counted every four days from day 7 to day 27 after transduction. A growth curve of OSK-SW480 cells was lower than all the other cells at both the day 15 (upper panel) and the day 27 (lower panel). The error bars indicate the SD. (n = 3).
(PDF)

Figure S4 Transduction of *OCT3/4*, *SOX2* and *KLF4* (OSK) into DLD-1 cells. A Mock (empty vector) or OSK was retrovirally transduced into DLD-1 cells (Mock-DLD-1, OSK-DLD-1, respectively). (A) The proliferation during a 72 hr period *in vitro*. A total of 3×10^5^ cells were plated on six-well plates on day seven and were counted on day 10. The number of OSK-DLD-1 cells was significantly lower than that of the parental DLD-1 (Wt-DLD-1) and Mock-DLD-1 cells (n = 3). The error bars indicate the SD. ^**^P<0.01, Dunnett's test. (B) The tumorigenicity in immunodeficient mice. A total of 1×10^5^ cells were subcutaneously injected into both flanks of immunodeficient nude mice on day 10. The tumor volume was measured eight weeks after injection. A summary of the tumor incidence is shown in Table S2. The volumes of tumors derived from the OSK-DLD-1 cells were significantly higher than those derived from Mock-DLD-1 cells. The red bars indicate the median tumor volume. ^*^P<0.05, N.S.: not significant, Dunnett's test. (C) Hoechst33342-efflux activity on day 10. The OSK-DLD-1 cells contained cells unlabeled by 5 µg/ml of Hoechst33342 with the co-administration of 50 µM of verapamil (VM). The V0-cells increased in the OSK-DLD-1 population. The V50-cells were obviously seen in OSK-DLD-1 culture. The V50-cells in OSK-DLD-1 were labeled by 5 µg/ml of Hoechst33342 with the co-administration of 250 µM of VM.
(PDF)

Figure S5 The phenotypical diversity in efflux activity of derivatives of OSK-V50 cells. FCM analysis for the Hoechst33342-efflux activity of the sorted cells after 23 days in culture. The M-V0 cells produced V0-cells and non-V0-cells. The M-nonV50 cells, which consisted of M-V0 and M-nonV0 populations, produced V0-cells and non-V0-cells, but not V50-cells (upper panel). The OSK-nonV50 cells containing some OSK-V0 cells produced V0-, non-V0- and only a few V50-cells. In contrast, the OSK-V50 cells produced V0-, non-V0-, V50-, non-V50- and V250-cells, which are unlabeled by Hoechst33342 in the presence of 250 µM of VM (lower panel). VM: verapamil.
(PDF)

Figure S6 The results of the dye efflux activity analysis of cultured cells dissociated from the tumors in serial transplantation experiments. The panel shows representative dot plots of the cells cultured for six to 12 days after dissociation. In the case of OSK-V50 cells, the V50 cells were serially observed in each of the sets of cultured cells. In contrast, V50 cells were not observed in the second cultured M-nonV50 cells. VM: verapamil.
(PDF)

Figure S7 The pathological findings of the tumors in serial transplantation experiments. The upper and lower panels represent the pathological findings for each series of tumors in each serial transplantation experiment. The OSK-V50 cells serially showed the same pathological features as those described in Figures 6A and 6B; there was cell diversity and glandular structures noted in HE staining, and cells were CK20- and CDX2-positive and CK7-negative in the immunostaining studies. Scale bars: 50 µm.
(PDF)

Figure S8 The characterization of the re-sorted V50 cells in serial transplantation experiments. (A) A schematic representation of the experiments. The V50 and nonV50 cells were re-sorted and collected from third cultured cells on day 9 after the dissociation of the second tumors by FACS sorting (n = 3). (B) The proliferation of re-sorted cells from the third cultured cells *in vitro*. A total of 3×10^5^ cells cultured for 12 to 14 days after sorting were seeded and counted 96 hr later. The number of cells was significantly lower in the re-sorted V50 cells than in the re-sorted nonV50 cells (n = 3). **P<0.01, t-test. (C) The morphologies of the re-sorted V50 and nonV50 cells. The day when each photograph was taken is indicated, and photographs were taken for each culture period after re-sorting. The re-sorted V50 cells exhibited morphological features similar to those of OSK-V50 cells, as shown in Figures 4A and 5B, resulting in an increase in cell diversity with time. Scale bars: 100 µm. (D) The pathological findings of tumors derived from re-sorted V50 cells in each independent experiment. The re-sorted V50 cells showed the same pathological features as those described in Figures 6A, 6B, 7E and S7; there was cell diversity and glandular structures observed by HE staining, and the immunostaining showed positive findings for CK20 and CDX2 and negative findings for CK7. Exp: experiment. Scale bars: 50 µm. (E) The results of the dye efflux activity analysis. The panel showed the data from three independent experiments subjected to a dye efflux activity analysis for the cells that were cultured for six to eight days after the dissociation of tumors derived from re-sorted V50 cells. VM: verapamil, Exp: experiment.
(PDF)

Table S1 Primer sequences used in qRT-PCR.
(PDF)

Table S2 Summary of tumor formation derived from transduced DLD-1 cells.
(PDF)

Video S1 Time-lapse imaging of M-V0 cells.
(MOV)

Video S2 Time-lapse imaging of OSK-V50 cells.
(MOV)

Methods S1 (PDF)
