# Supplementary figures and images for: Induction of Cancer Stem Cell Properties in Colon Cancer Cells by Defined Factors
Source: PLoS One. 2014 Jul 9;9(7):e101735. doi: 10.1371/journal.pone.0101735 (PMC4090165; doi:10.1371/journal.pone.0101735)

**Figure S1**

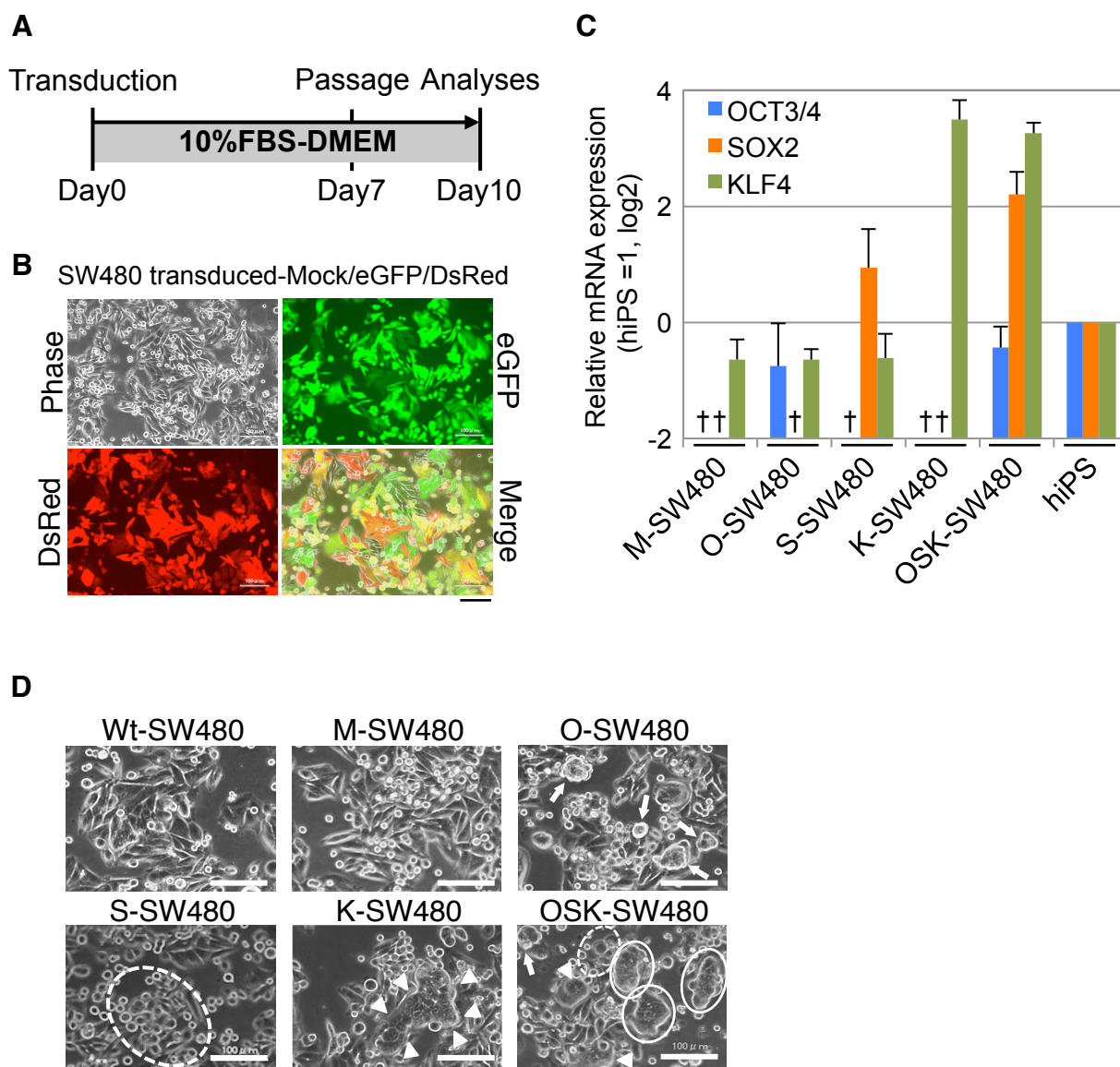

Supplement: Figure S1 — Transduction of OCT3/4, SOX2, KLF4 or a mixture of the three factors (OSK) in SW480 cells. (A) A schematic representation of this study. (B) The transduction efficiency in SW480 cells. Cells were retrovirally transduced with a mixture of three factors; eGFP, DsRed and Mock (empty vector). Almost all cells expressed at least one the transduced genes. Around half of the cells expressed both eGFP and DsRed. Scale bar: 100 µm. (C) qRT-PCR of OCT3/4, SOX2 and KLF4 in transduced SW480 cells using primers common for both endogenous and exogenous transcripts. The mRNA expression levels were normalized to those of GAPDH. The relative expression levels compared to those to human iPSCs are shown. All the transduced genes were obviously expressed. The error bars indicate the SD (n = 3). † : not detected. (D) The morphology of the transduced SW480 cell lines. The transduction of each individual factor led to distinct morphological changes (arrow, dotted circle or arrow head). Simultaneous transduction of the three factors also led to specific morphologic features (lined circle). Parental cells (Wt-SW480) and M-SW480 cells predominantly consisted of spindle-shaped cells and small round-shaped cells. In the O-SW480 culture, globular clusters consisting of cells with unclear edges appeared. In the S-SW480 cultures, the number of round-shaped cells was increased. In K-SW480 cultures, colonies consisting of cells with slightly unclear edges appeared. In the OSK-SW480 cultures, flat-mounted colonies consisting of cells with unclear edges appeared, and similar morphologies were also seen as were noted in the other cells. Scale bars: 100 µm. (PDF) [file pone.0101735.s001.pdf]

Figure S2

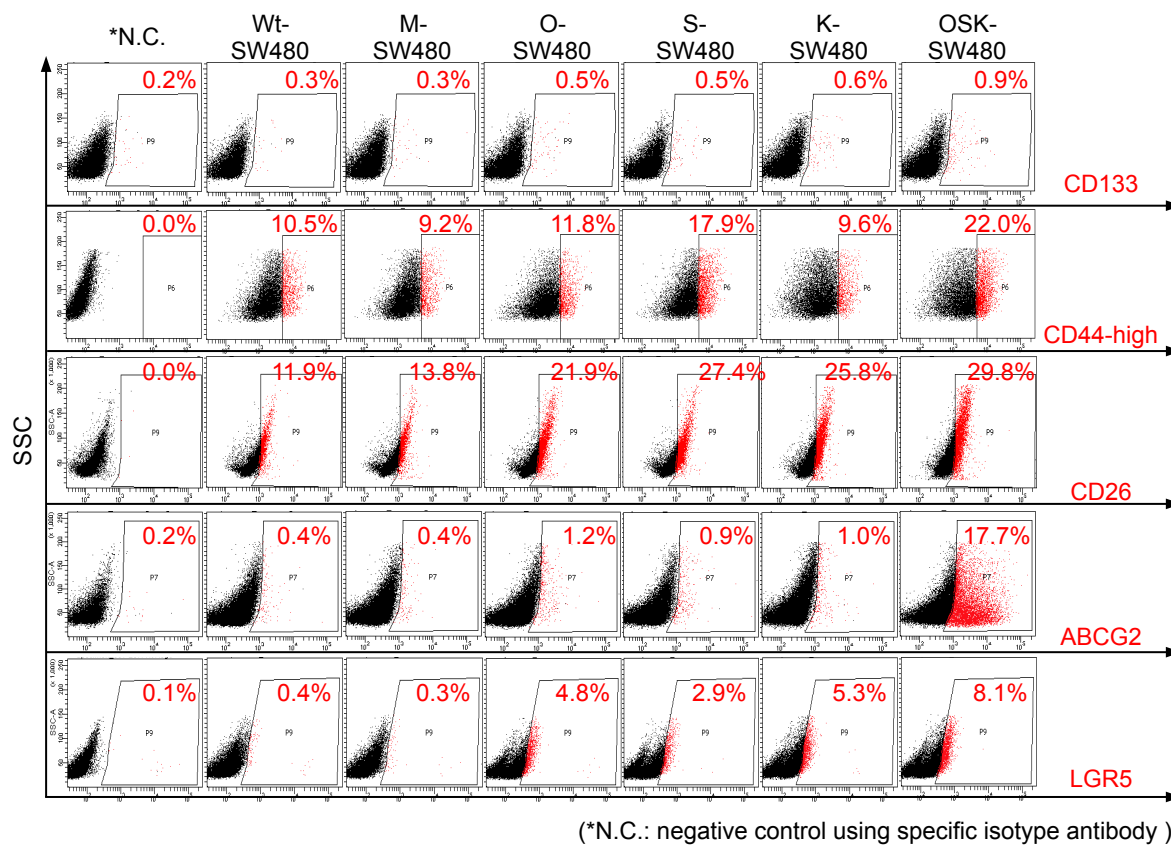

Supplement: Figure S2 — The results of the flow cytometric (FCM) analysis of the CSC marker protein expression in transduced SW480 cells. The panel shows representative dot plots of the cells expressing CD133, CD44, CD26, ABCG2 and LGR5 in the transduced SW480 cells. (PDF) [file pone.0101735.s002.pdf]

**Figure S3**

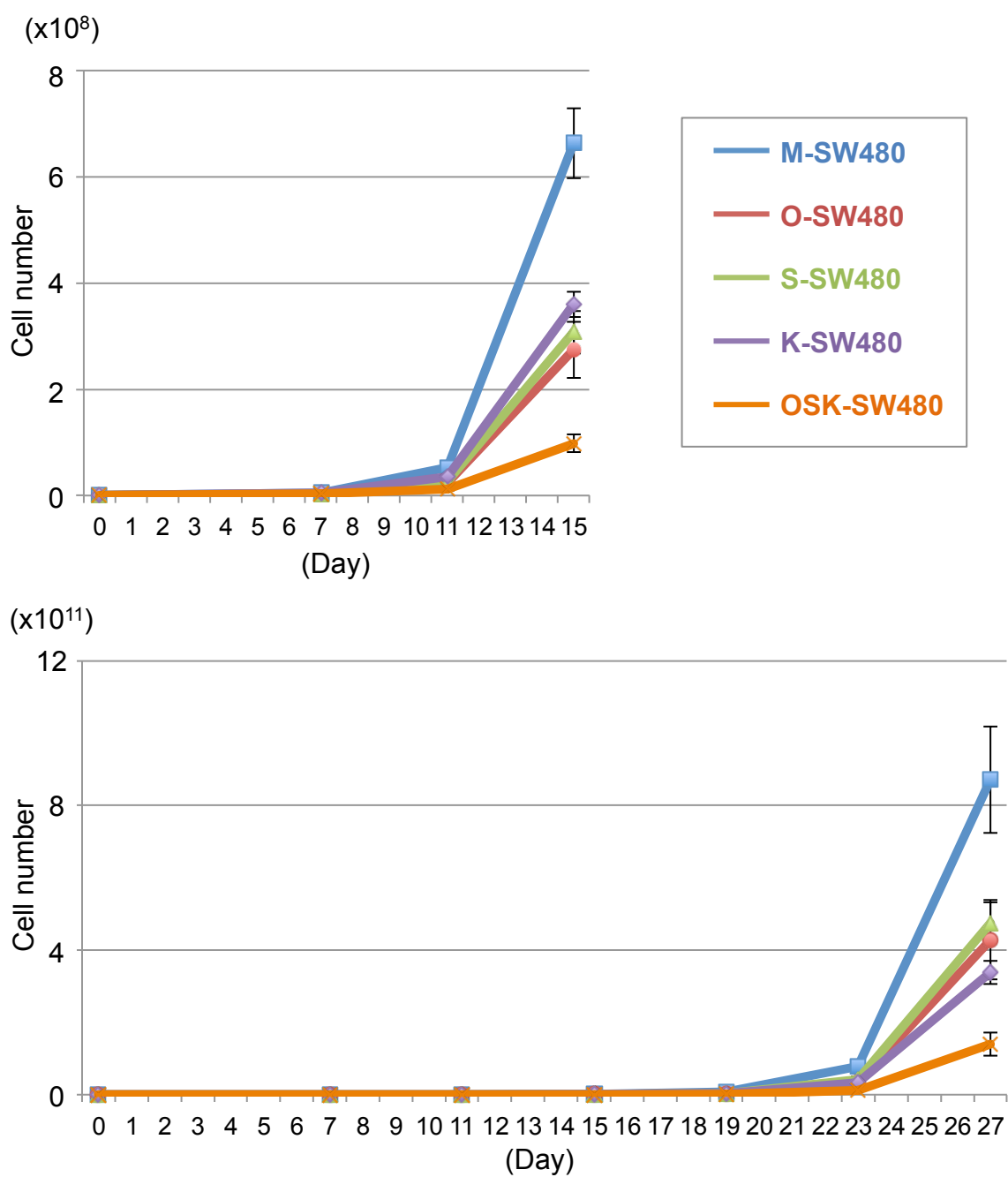

Supplement: Figure S3 — Cell proliferation of transduced SW480 cells in vitro. The cell number of transduced SW480 cells was counted every four days from day 7 to day 27 after transduction. A growth curve of OSK-SW480 cells was lower than all the other cells at both the day 15 (upper panel) and the day 27 (lower panel). The error bars indicate the SD. (n = 3). (PDF) [file pone.0101735.s003.pdf]

**Figure S4**

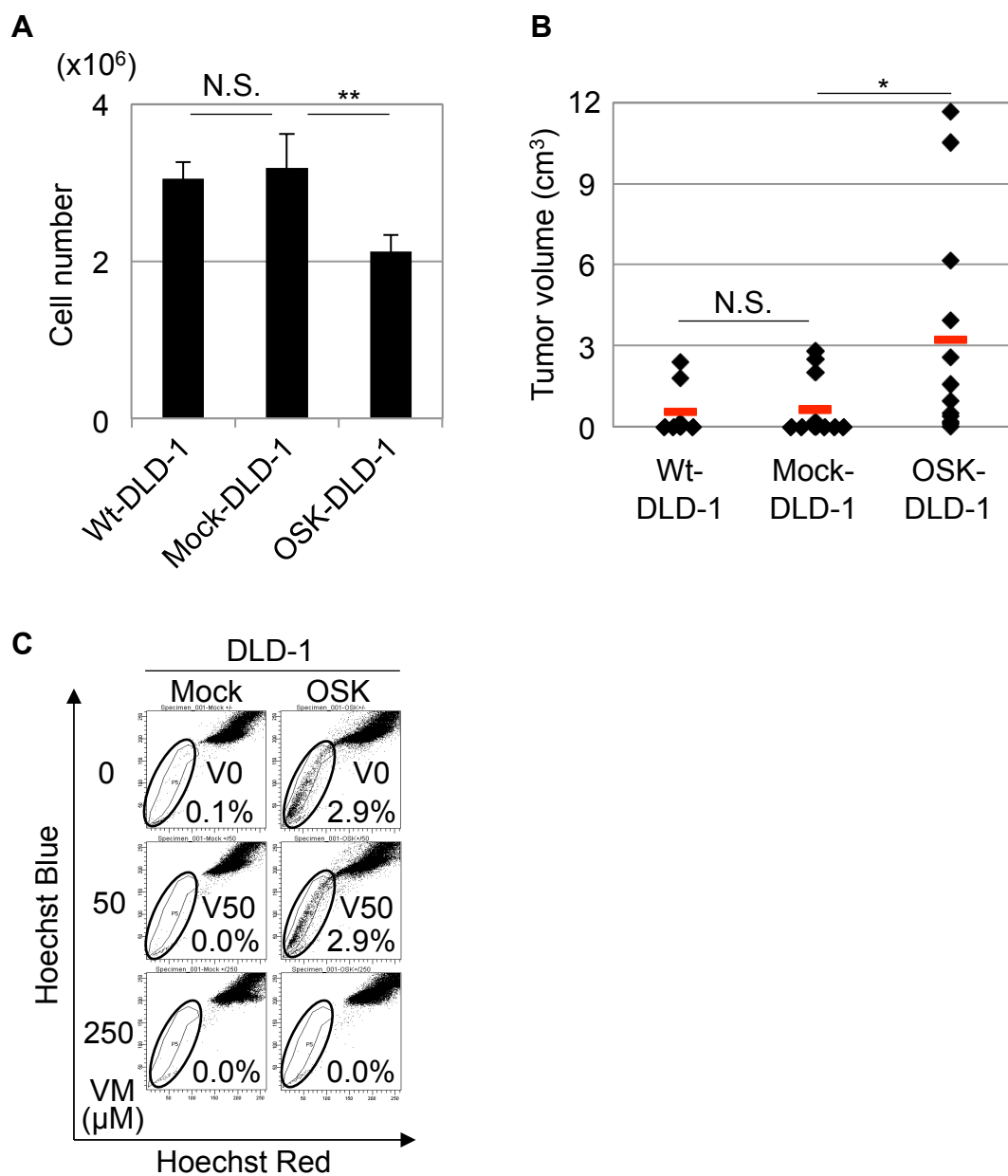

Supplement: Figure S4 — Transduction of OCT3/4, SOX2 and KLF4 (OSK) into DLD-1 cells. A Mock (empty vector) or OSK was retrovirally transduced into DLD-1 cells (Mock-DLD-1, OSK-DLD-1, respectively). (A) The proliferation during a 72 hr period in vitro. A total of 3×10^5^ cells were plated on six-well plates on day seven and were counted on day 10. The number of OSK-DLD-1 cells was significantly lower than that of the parental DLD-1 (Wt-DLD-1) and Mock-DLD-1 cells (n = 3). The error bars indicate the SD. **P<0.01, Dunnett's test. (B) The tumorigenicity in immunodeficient mice. A total of 1×10^5^ cells were subcutaneously injected into both flanks of immunodeficient nude mice on day 10. The tumor volume was measured eight weeks after injection. A summary of the tumor incidence is shown in Table S2. The volumes of tumors derived from the OSK-DLD-1 cells were significantly higher than those derived from Mock-DLD-1 cells. The red bars indicate the median tumor volume. *P<0.05, N.S.: not significant, Dunnett's test. (C) Hoechst33342-efflux activity on day 10. The OSK-DLD-1 cells contained cells unlabeled by 5 µg/ml of Hoechst33342 with the co-administration of 50 µM of verapamil (VM). The V0-cells increased in the OSK-DLD-1 population. The V50-cells were obviously seen in OSK-DLD-1 culture. The V50-cells in OSK-DLD-1 were labeled by 5 µg/ml of Hoechst33342 with the co-administration of 250 µM of VM. (PDF) [file pone.0101735.s004.pdf]

Figure S5

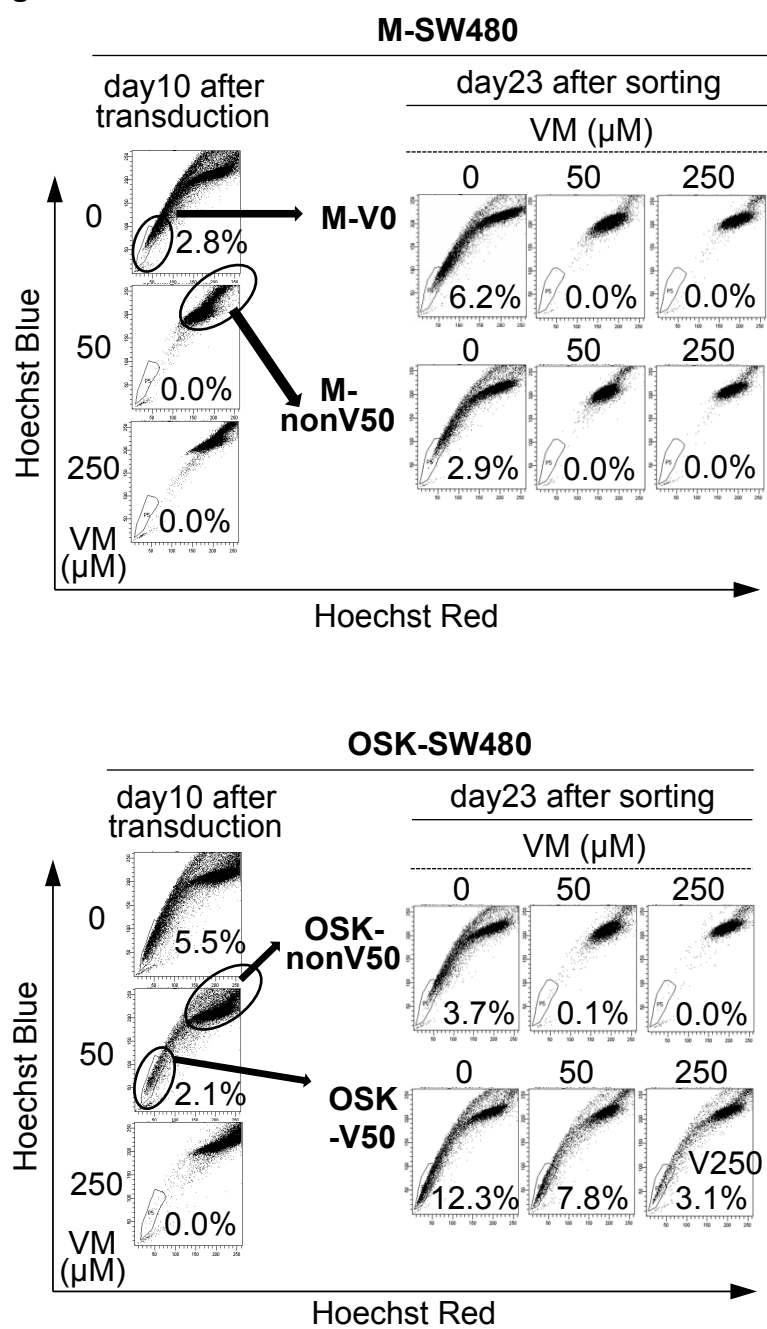

Supplement: Figure S5 — The phenotypical diversity in efflux activity of derivatives of OSK-V50 cells. FCM analysis for the Hoechst33342-efflux activity of the sorted cells after 23 days in culture. The M-V0 cells produced V0-cells and non-V0-cells. The M-nonV50 cells, which consisted of M-V0 and M-nonV0 populations, produced V0-cells and non-V0-cells, but not V50-cells (upper panel). The OSK-nonV50 cells containing some OSK-V0 cells produced V0-, non-V0- and only a few V50-cells. In contrast, the OSK-V50 cells produced V0-, non-V0-, V50-, non-V50- and V250-cells, which are unlabeled by Hoechst33342 in the presence of 250 µM of VM (lower panel). VM: verapamil. (PDF) [file pone.0101735.s005.pdf]

Figure S6

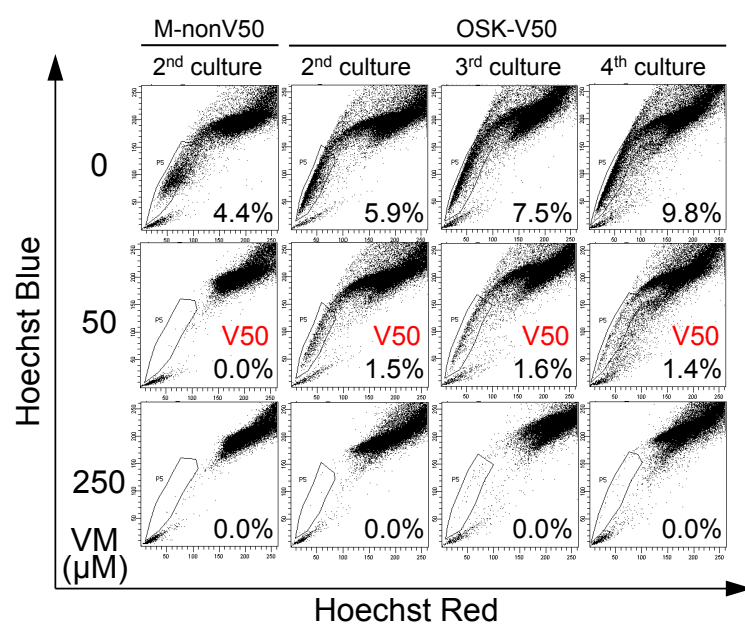

Supplement: Figure S6 — The results of the dye efflux activity analysis of cultured cells dissociated from the tumors in serial transplantation experiments. The panel shows representative dot plots of the cells cultured for six to 12 days after dissociation. In the case of OSK-V50 cells, the V50 cells were serially observed in each of the sets of cultured cells. In contrast, V50 cells were not observed in the second cultured M-nonV50 cells. VM: verapamil. (PDF) [file pone.0101735.s006.pdf]

**Figure S7**

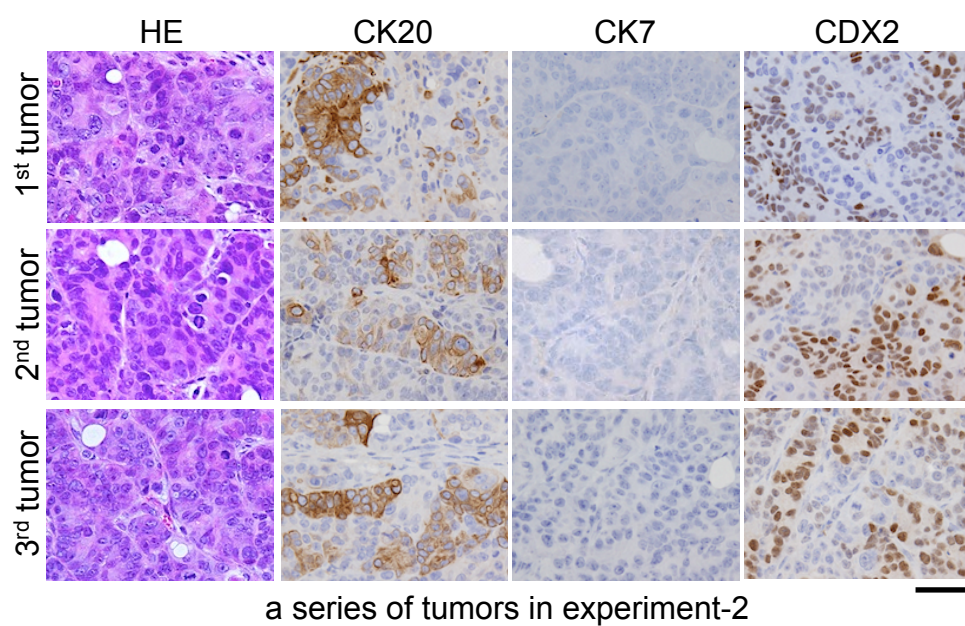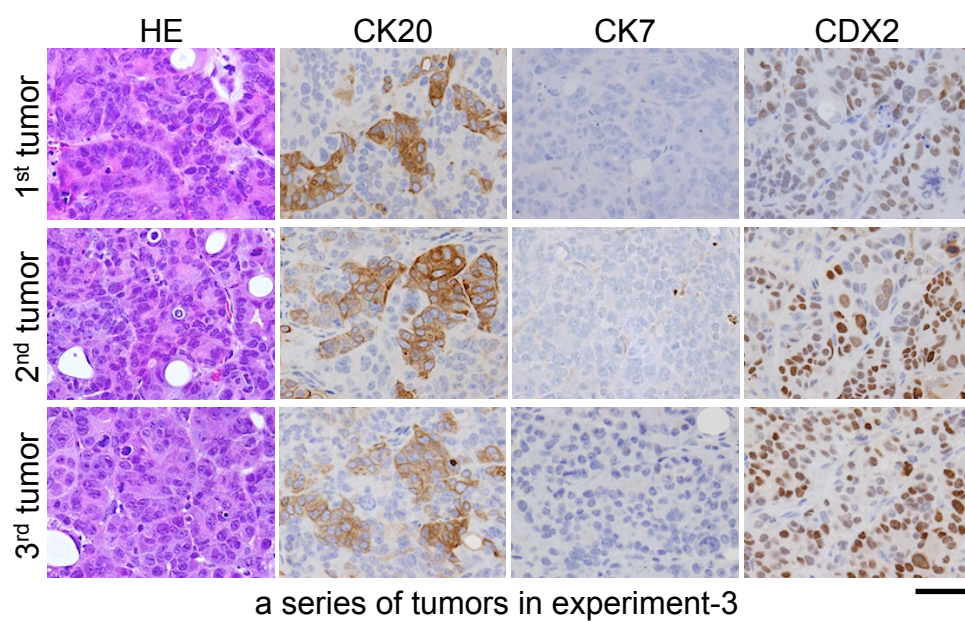

Supplement: Figure S7 — The pathological findings of the tumors in serial transplantation experiments. The upper and lower panels represent the pathological findings for each series of tumors in each serial transplantation experiment. The OSK-V50 cells serially showed the same pathological features as those described in Figures 6A and 6B; there was cell diversity and glandular structures noted in HE staining, and cells were CK20- and CDX2-positive and CK7-negative in the immunostaining studies. Scale bars: 50 µm. (PDF) [file pone.0101735.s007.pdf]

Figure S8

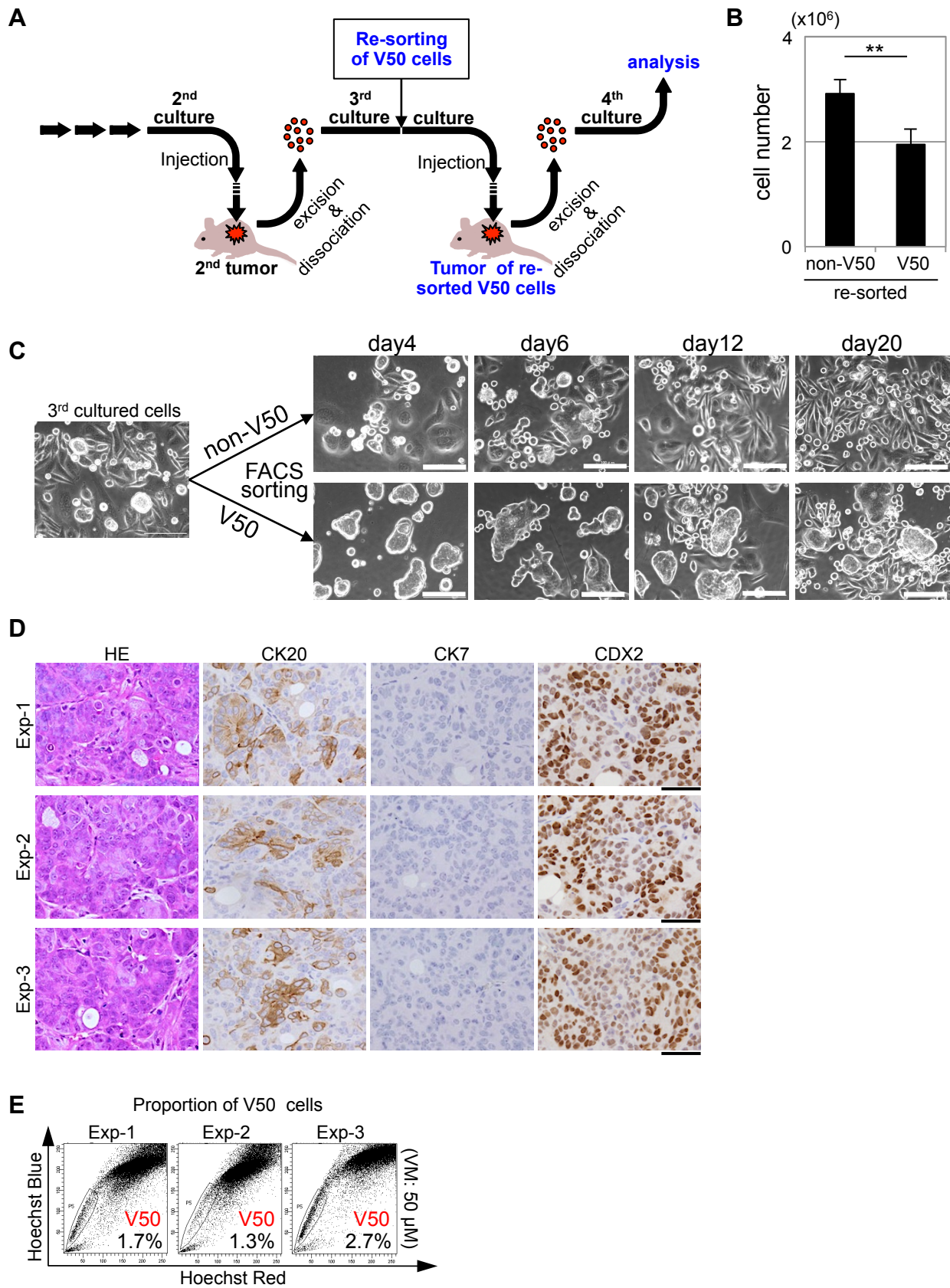

Supplement: Figure S8 — The characterization of the re-sorted V50 cells in serial transplantation experiments. (A) A schematic representation of the experiments. The V50 and nonV50 cells were re-sorted and collected from third cultured cells on day 9 after the dissociation of the second tumors by FACS sorting (n = 3). (B) The proliferation of re-sorted cells from the third cultured cells in vitro. A total of 3×10^5^ cells cultured for 12 to 14 days after sorting were seeded and counted 96 hr later. The number of cells was significantly lower in the re-sorted V50 cells than in the re-sorted nonV50 cells (n = 3). **P<0.01, t-test. (C) The morphologies of the re-sorted V50 and nonV50 cells. The day when each photograph was taken is indicated, and photographs were taken for each culture period after re-sorting. The re-sorted V50 cells exhibited morphological features similar to those of OSK-V50 cells, as shown in Figures 4A and 5B, resulting in an increase in cell diversity with time. Scale bars: 100 µm. (D) The pathological findings of tumors derived from re-sorted V50 cells in each independent experiment. The re-sorted V50 cells showed the same pathological features as those described in Figures 6A, 6B, 7E and S7; there was cell diversity and glandular structures observed by HE staining, and the immunostaining showed positive findings for CK20 and CDX2 and negative findings for CK7. Exp: experiment. Scale bars: 50 µm. (E) The results of the dye efflux activity analysis. The panel showed the data from three independent experiments subjected to a dye efflux activity analysis for the cells that were cultured for six to eight days after the dissociation of tumors derived from re-sorted V50 cells. VM: verapamil, Exp: experiment. (PDF) [file pone.0101735.s008.pdf]
