# Supplementary material for: Induction of Cancer Stem Cell Properties in Colon Cancer Cells by Defined Factors
Source: PLoS One. 2014 Jul 9;9(7):e101735. doi: 10.1371/journal.pone.0101735 (PMC4090165; doi:10.1371/journal.pone.0101735)
Supplement: Table S1 — Primer sequences used in qRT-PCR. (PDF) [file pone.0101735.s009.pdf]

Table S1

Primer sequences used in qRT-PCR

Target gene	Sequence (5' to 3')
hOCT3/4(endo and trans)	CCC CAG GGC CCC ATT TTG GTA CC
	ACC TCA GTT TGA ATG CAT GGG AGA GC
hSOX2(endo and trans)	TTC ACA TGT CCC AGC ACT ACC AGA
	TCA CAT GTG TGA GAG GGG CAG TGT GC
hKLF4(endo and trans)	CAT GCC AGA GGA GCC CAA GCC AAA GAG GGG
	CGC AGG TGT GCC TTG AGA TGG GAA CTC TTT
CD44	AGA AGG TGT GGG CAG AAG AA
	AAA TGC ACC ATT TCC TGA GA
CD26	CAA ATT GAA GCA GCC AGA CA
	CAC ACT TGA ACA CGC CAC TT
CD133	TGG GGC TGC TGT TTA TTA TTC T
	TGC CAC AAA ACC ATA GAA GAT G
ALDH1	TCC TGG TTA TGG GCC TAC AG
	CTG GCC CTG GTG GTA GAA TA
ABCG2	AGC TGC AAG GAA AGA TCC AA
	TCC AGA CAC ACC ACG GAT AA
LGR5	GAT GTT GCT CAG GGT GGA CT
	TTT CCC GCA AGA CGT AAC TC
GAPDH	ACC ACA GTC CAT GCC ATC AC
	TCC ACC ACC CTG TTG CTG TA
