# Supplementary material for: Induction of Cancer Stem Cell Properties in Colon Cancer Cells by Defined Factors
Source: PLoS One. 2014 Jul 9;9(7):e101735. doi: 10.1371/journal.pone.0101735 (PMC4090165; doi:10.1371/journal.pone.0101735)
Supplement: Table S2 — Summary of tumor formation derived from transduced DLD-1 cells. (PDF) [file pone.0101735.s010.pdf]

Table S2

Summary of tumor formation derived from transduced DLD-1 cells

	Tumor formation
	Injected cell number
Cell name	1 $x10^5$
Wt-DLD-1	50% (4/8)
Mock-DLD-1	50% (6/12)
OSK-DLD-1	91.6% (11/12)
