# Supplementary material for: Induction of Cancer Stem Cell Properties in Colon Cancer Cells by Defined Factors
Source: PLoS One. 2014 Jul 9;9(7):e101735. doi: 10.1371/journal.pone.0101735 (PMC4090165; doi:10.1371/journal.pone.0101735)
Supplement: Methods S1 — (PDF) [file pone.0101735.s013.pdf]

Supplementary Methods

Retroviral Infection

For retroviral infection, we modified the previously described methods [1,2], and used pMXs vectors with the same constructs as those described in a previous report[2]. PLAT-A packaging cells were plated at 4.0×10^5 cells per six-well dish, and were incubated overnight. On the following day, the cells were transfected with pMXs vectors[3] using the Fugene6 transfection reagent (Roche, Basel, Switzerland). Twenty-four hours after transfection, the medium was replaced with new medium, which was collected after another 24 hr as the virus-containing supernatant. The virus-containing supernatants were filtered through a 0.45 μm pore-size filter and supplemented with a concentration of 4 μg/ml polybrene (Nacalai Tesque, Kyoto, Japan). Equal amounts of supernatants containing each of the retroviruses were mixed, transferred to the cancer cell line dish that was prepared the previous day, and incubated overnight. Another 24 hr after infection, the virus-containing medium was replaced with fresh medium without retroviruses.

Time-lapse observation

The cells were filmed every 6 hr for 42hr by a cell culture observation system incubator (BioStation CT, Nikon, Tokyo, Japan) from 24 hr after seeding. The time-lapse images were analyzed by a CL-Quant analysis software (Nikon).

Supplementary Reference

1. Takahashi K, Yamanaka S (2006) Induction of pluripotent stem cells from mouse embryonic and adult fibroblast cultures by defined factors. Cell 126: 663-676.
2. Takahashi K, Tanabe K, Ohnuki M, Narita M, Ichisaka T, et al. (2007) Induction of pluripotent stem cells from adult human fibroblasts by defined factors. Cell 131: 861-872.
3. Kitamura T, Koshino Y, Shibata F, Oki T, Nakajima H, et al. (2003) Retrovirus-mediated gene transfer and expression cloning: powerful tools in functional genomics. Experimental hematology 31: 1007-1014.
